# A transcriptomics data-driven gene space accurately predicts liver cytopathology and drug-induced liver injury

**DOI:** 10.1038/ncomms15932

**Published:** 2017-07-03

**Authors:** Pekka Kohonen, Juuso A. Parkkinen, Egon L. Willighagen, Rebecca Ceder, Krister Wennerberg, Samuel Kaski, Roland C. Grafström

**Affiliations:** 1 Institute of Environmental Medicine, Karolinska Institutet, Nobels väg 13, Box 210, SE-17177 Stockholm, Sweden; 2 Helsinki Institute for Information Technology HIIT, Department of Computer Science, Aalto University, Konemiehentie 2, P.O. Box 15400, 00076 Aalto, Finland; 3 Department of Bioinformatics—BiGCaT, Maastricht University, Universiteitssingel 50, P.O. Box 616, UNS 50 Box19, NL-6200 MD Maastricht, The Netherlands; 4 Institute for Molecular Medicine Finland, FIMM, University of Helsinki, Tukholmankatu 8, P.O. Box 20, FI-00014 Helsinki, Finland; 5 Helsinki Institute for Information Technology HIIT, Department of Computer Science, University of Helsinki, Gustaf Hällströmin katu 2b, P.O. Box 68, FI-00014 Helsinki, Finland

## Abstract

Predicting unanticipated harmful effects of chemicals and drug molecules is a difficult and costly task. Here we utilize a ‘big data compacting and data fusion’—concept to capture diverse adverse outcomes on cellular and organismal levels. The approach generates from transcriptomics data set a ‘predictive toxicogenomics space’ (PTGS) tool composed of 1,331 genes distributed over 14 overlapping cytotoxicity-related gene space components. Involving ∼2.5 × 10^8^ data points and 1,300 compounds to construct and validate the PTGS, the tool serves to: explain dose-dependent cytotoxicity effects, provide a virtual cytotoxicity probability estimate intrinsic to omics data, predict chemically-induced pathological states in liver resulting from repeated dosing of rats, and furthermore, predict human drug-induced liver injury (DILI) from hepatocyte experiments. Analysing 68 DILI-annotated drugs, the PTGS tool outperforms and complements existing tests, leading to a hereto-unseen level of DILI prediction accuracy.

## Abstract (web summary)

Predicting the hepatotoxic effects of new drugs is still a challenge. Using toxicogenomics data, the authors here define a predictive toxicogenomic space (PTGS), the component gene space capturing dose-dependent cytotoxicity, and demonstrate that it can be used to accurately predict drug-induced liver pathology, including human drug-induced liver injury from *in vitro* data.

Products and compounds to be placed in products undergo safety testing to variable levels of depth and complexity. Even highly regulated and rigorous testing practices unfortunately still fail in detecting inherent toxicological properties, of which some effects become evident only after exposure to a marketed product^1^,^2^,^3^. For the drug developing pharmaceutical industry, severe drug-induced liver injury (DILI) remains an enormous problem, as its occurrence is often not predicted^3^,^4^,^5^,^6^.

Systems biology-based assays relying upon quantitative mechanistic information are increasingly envisaged as cornerstones of future safety evaluation of drugs and chemicals^1^,^7^,^8^,^9^,^10^,^11^. Accordingly, various modelling approaches have analysed ‘omics data sets to generate biomarker signatures or to characterize mechanisms of toxicity at a system-wide level, but suffer from the high dimensionality of omics data relative to sample number as well as problems in scaling across experimental systems (for example, hepatocyte cultures to liver) or species (for example, rat to human)^11^,^12^,^13^,^14^,^15^,^16^. Complicating predictive biomarker discovery, different genes and gene families will unlikely have similar dose response, so dose-dependent transitions will influence the classification of (toxic) modes-of-action^17^,^18^. Addressing the issue, the adverse outcome pathways (AOP) concept was designed to facilitate the use of modern human-specific *in vitro* models to understand toxicity and disease pathways at multiple levels of biological organization^19^,^20^. Embracing the complete chain of events from the first compound-induced molecular cellular changes to influence adversity on cellular, organ, individual and even population levels, the AOP concept has been incorporated into *in vitro* tests for an accepted replacement of animal experiments^20^. Large-scale consortia and projects, for example, Tox21, ToxCast and SEURAT/EUToxRisk, address the issue by complementing the traditional structure-based analysis with developing ‘new approach methodologies’ for safety prediction inspired by the AOP concept, including transcriptomics assays^10^,^21^,^22^,^23^,^24^.

The US Broad Institute Connectivity Map (CMap) data set has thousands of gene expression profiles of mostly FDA approved drugs and has been used to connect small molecules, genes and diseases (‘connectivity mapping’) to define biologically similar compounds, including for the purpose of identifying toxic modes of action^11^,^15^,^25^,^26^,^27^,^28^,^29^. The US National Cancer Institute (NCI) 60 tumour cell line screen includes results on GI_50_ (50% growth inhibition), total growth inhibition (TGI), and LC_50_ (50% lethal concentration) for many compounds tested in the major CMap cell lines^30^. A number of screening assays have been developed to explore possible mechanisms of DILI with the ultimate aim of predicting clinical exposure levels of concern^6^,^31^,^32^. The US FDA Liver Toxicity Knowledge Base (LTKB) is a centralized resource for drug information related to liver damage^33^. The Open ‘Toxicogenomics Project-Genomics Assisted Toxicity Evaluation system' (TG-GATEs) constitutes a resource that spans both *in vitro* and *in vivo* analyses of 158 potentially hepatotoxic compounds^16^. On the basis of these data sources we decided to test the hypothesis that a predictive set of toxicogenomics-relevant changes would lie within the large space of chemically induced transcriptomic alterations of the CMap, most of which could potentially be extracted and validated via bioinformatics processing of cytotoxicity effects and gene profiling results. As the CMap database generally has a single concentration per compound and cell line, this idea included the *a-priori* assumption that dose–response relationships should potentially be captured by the cross-compound potency–response relationships. We further selected hepatocellular toxicity prediction as the initial target of this approach, the ultimate aim being to predict human organ-level clinical toxicity using preclinical data. This objective included to assess if the approach could serve to complement existing DILI tests^6^,^32^.

Here we describe a data compacting modelling approach^34^,^35^, and apply it to the gene space of the CMap. Fusing the resulting component space with cytotoxicity data from the NCI-60 tumour cell line screen generates a predictive toxicogenomics space (PTGS). Enrichment analyses relative to pathways and gene regulators, cell culture experiments, compound structure-based analyses and assessment of the large independent data source constituted by the TG-GATEs demonstrates that PTGS captures all studied liver pathological changes observed in rats. Moreover, PTGS enables high-fidelity prediction of human DILI from hepatocyte-derived toxicogenomics data.

## Results

### Modelling for generating a Predictive Toxicogenomic Space

**Figure 1:**
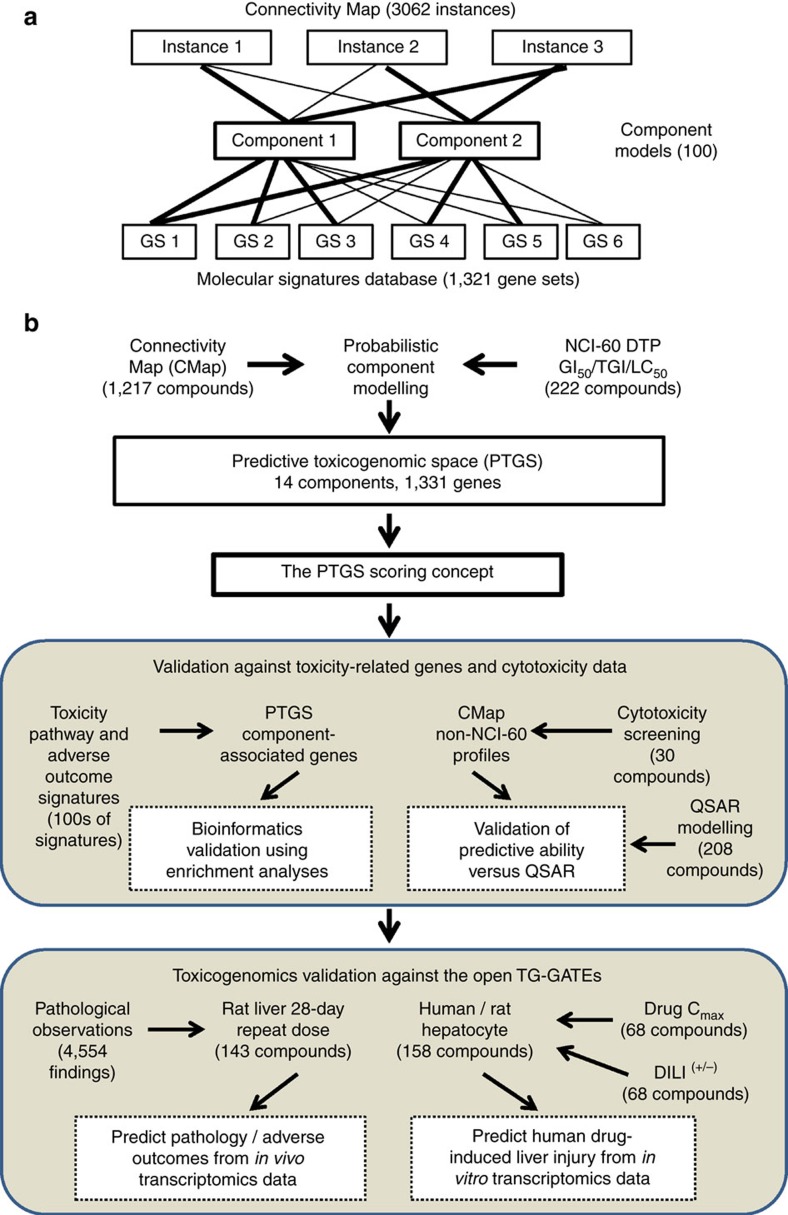
Generating the Predictive Toxicogenomics Space (PTGS) concept. (**a**) The probabilistic component modelling leading to the PTGS scoring concept utilized latent Dirichlet allocation. This unsupervised method uncovers common themes that describe collections of profiles, seeking associations between compound treatments (‘instances’) and differential expression of gene sets, leading to data reduction and discovery of components that can be used to quantitatively classify new gene expression profiles. (**b**) Probabilistic modelling of transcriptomics and cytotoxicity data from exposed cells was used to identify specific component models representing mechanistic aspects of the responses and genes activated by the treatments. Scores derived either from the models or the gene set encapsulated by the PTGS serve to predict a variety of types of dose-dependent cytotoxicity effects; the analysis steps are presented in detail in Supplementary Fig. 1. Validation of the PTGS scoring concept encompassed: bioinformatics-driven assessment of the component-associated genes relative to genes known as cytotoxicity-related, generation of cellular cytotoxicity screening data for comparison of the omics-based PTGS relative to quantitative structure-activity relationships (QSAR) analysis, and finally, assessment of the *in vitro* to *in vivo* extrapolation applicability of the PTGS in two ways against the Open 'Toxicogenomics Project-Genomics Assisted Toxicity Evaluation system' (TG-GATEs), that is, for prediction of histopathology of rats subjected to repeat dose-toxicity studies, and for prediction of human drug-induced liver injury from human and rat hepatocytes. Numbers of compounds assessed within each omics data set used to establish the PTGS and to validate the concept are indicated.

The PTGS was defined with probabilistic component modelling of the combined CMap and NCI-60 data, as the minimally sized component gene space that captured dose-dependent cytotoxicity within the complete data set. Modelling of the CMap transcriptomics response was done in a two-step semi-supervised manner; performing unsupervised component modelling on the whole CMap data set and subsequently using the component models and the NCI-60 cytotoxicity data to build supervised models. Gene sets that represent the components were then derived and applied as a basis for predictive scoring; Fig. 1 and Supplementary Fig. 1 depict the overall analysis and validation strategies that generated the PTGS. The protocol extracted and reduced the number of data points, compounds and genes. Positive concentration-dependent data indicated which CMap measurement instances had been produced at a concentration inducing at least 50% growth inhibition, and therefore reflected a potentially cytotoxic response (Supplementary Fig. 2).

**Figure 2:**
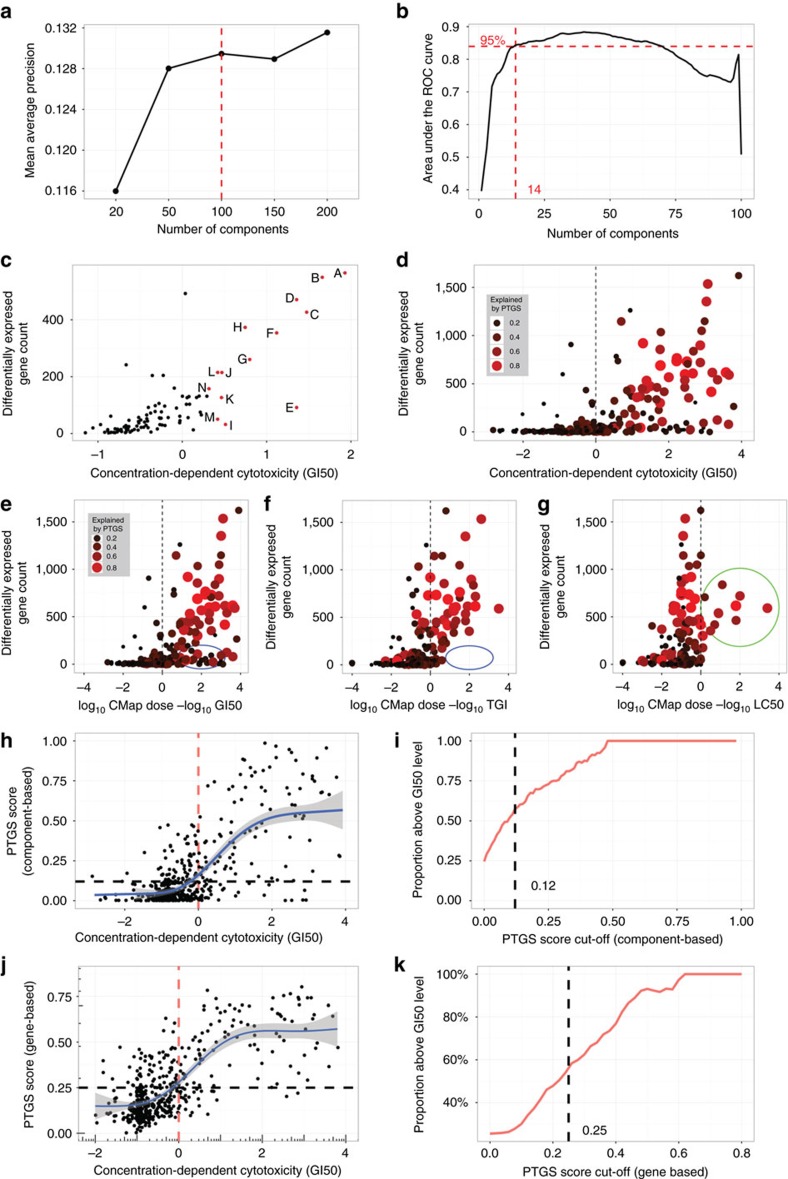
Generating the PTGS and establishing the cytotoxicity-scoring concept. (**a**) Selecting the number of probabilistic components to retrieve as many biologically significant associations with as few components as possible. (**b**) Selecting an optimal size of the PTGS based on cytotoxicity-predictive performance relative to the NCI-60 data. (**c**) The 14 PTGS components (labelled) ranked based on their probability-weighted mean concentration-dependent cytotoxicity values (that is, log_10_CMap–log_10_GI_50_ concentration) versus the number of associated genes. (**d**) Correlation of the number of differentially expressed genes with the concentration-dependent cytotoxicity. Colour and size indicate amount of transcriptional variation explained by the PTGS that is, the component-based score (*n*=492). (**e**,**f**) Instances with a small number of differentially expressed genes tend to have cytotoxicity below the TGI level (blue oval), whereas (**g**) compounds profiled at cell-killing doses (>LC_50_) show greater differences (green circle). (**h**) Analysis of component-based PTGS scores versus concentration-dependent cytotoxicity was used to determine (**i**) a cut-off, plotted here against the proportion of instances above the GI_50_-level. Dashed red line indicates the threshold at the GI_50_-level and the dashed black line the cut-off at 0.12 when ∼50% of CMap instances are above GI_50_. (**j**) The gene-based scoring, based on the proportion of active PTGS-related genes, was evaluated similarly. (**k**) The cut-off was set at 25% (cf. Supplementary Figs 4 and 5, for data see Supplementary Data 1).

The modelling approach decomposed the entire pre-processed CMap data, consisting of 3062 instances (an instance represents a chemical treatment of one cell line), to 100 partially overlapping and non-orthogonal components (Fig. 2a). Superimposing the NCI-60 data enabled integrating 222 CMap compounds and 492 instances, measured over a ∼10^6^-fold potency range relative to GI_50_. This crossover data set permitted the selection of an optimally sized set of the 14 most cytotoxicity-associated components, which defined the PTGS (Fig. 2b,c). With area under the ROC curve (AUC) peaking at 40 components, these fourteen components give 95% of the highest AUC value for predicting whether cytotoxicity was above the GI_50_-level. Most of the components were proportionally active in all cell lines, suggesting that they capture generalizable cytotoxicity-related responses (Supplementary Fig. 3a). Hierarchical clustering of the PTGS revealed clustering of the components into one group comprising a majority of the components, another less distinct cluster (including E and K), and one outlier component (L), demonstrating that most of the components had overlapping gene activities (Supplementary Fig. 3b).

### Defining a cytotoxicity scoring concept from the PTGS

A PTGS-based scoring concept was defined based on the premise that activation of any of the PTGS components indicated dose-dependent cytotoxicity (Fig. 2, Supplementary Figs 4 and 5 and Supplementary Data 1). The cytotoxicity effects of the compounds correlated with the transcriptional variation (Pearson correlation is 0.69; *P*-value <2.2 × 10^−16^, Fig. 2c,d). The 14 components overall responded over a wide dose-range and, as expected, primarily became active at or above the GI_50_-dose (Fig. 2d–g). The PTGS therefore covered instances with varying numbers of differentially expressed genes and toxicities. Some CMap instances represented toxicities above the TGI level (Fig. 2e,f); such instances tended to have many differentially expressed genes (Fig. 2f) and highly active components A-C, D and F-H (Supplementary Figs 4 and 5a). On the other hand, instances belonging to the smaller cluster and components E, K, I and M tended to be active at around the GI_50_ growth-inhibitory level and displayed smaller numbers of differentially expressed genes (Fig. 2c,e,g; Supplementary Fig. 5b). A low number of instances that reflected cell-killing doses, that is, LC_50_, (Fig. 2g; Supplementary Fig. 5c) were also covered by the PTGS and components A-C in particular. A PTGS scoring concept for ranking compounds for probability of cytotoxicity was thereafter defined, that is, being the sum of the contributions of the 14 components relative to the other 86 components; for the calculation formula, see Materials and Methods (Fig. 2h). The score served to predict whether an instance was measured at a concentration above GI_50_, employing a decision-threshold designed to maximize sensitivity versus specificity (Fig. 2i).

The gene alterations corresponding to the PTGS components were next assessed and applied to cytotoxicity scoring (Fig. 2c,j and k). Overall 1331 genes associated to PTGS (listed in Supplementary Data 2). In analogy to the components, the PTGS-associated genes exhibited a dose–response relationship that could be used to predict with similar accuracy whether an instance was measured at a level above GI_50_. The composite of these results confirmed that the PTGS scoring constitutes a tool for predicting cytotoxicity over a wide range of concentrations and corresponding gene alterations.

### PTGS captures diverse cytopathological changes

**Figure 3:**
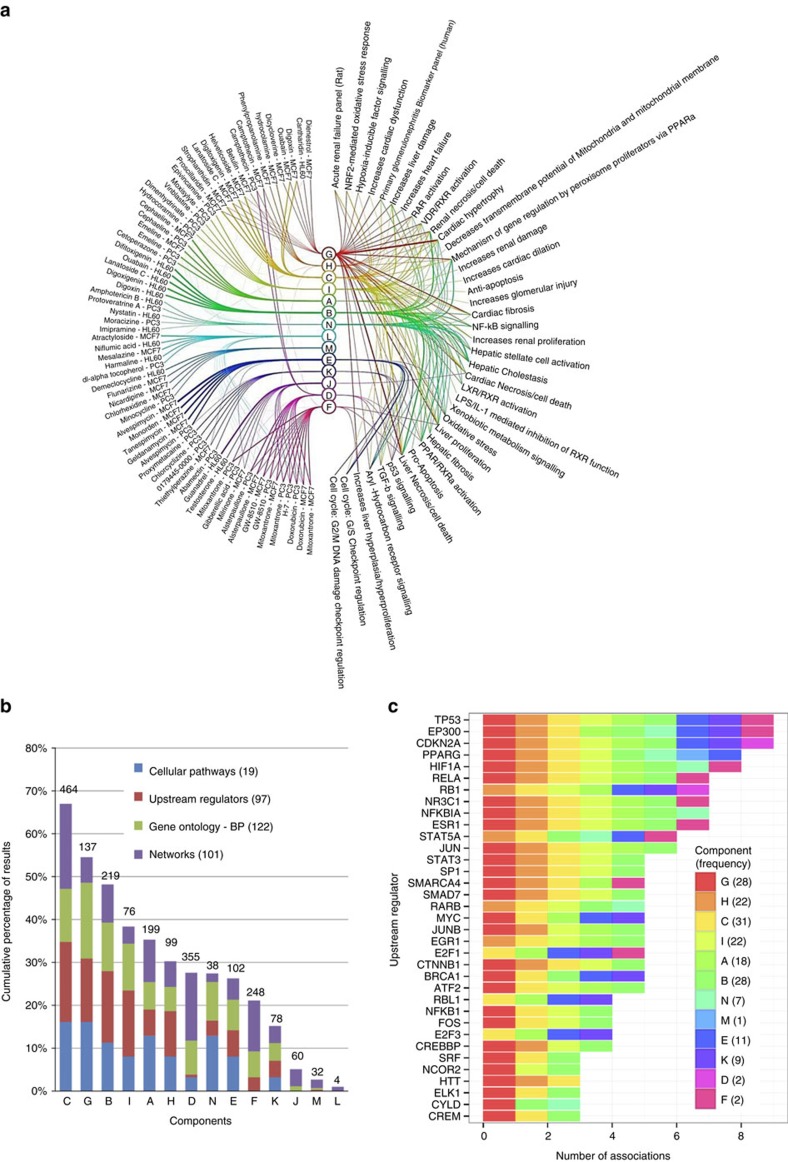
Validation of the PTGS using gene set enrichment analysis. (**a**) ‘Eye diagram’ showing the associations between the genes associated with the 14 PTGS components (middle, colour) and the top 5 CMap instances (left) and overrepresented toxicological functions (right). Line widths indicate association strengths. The components have been sorted according to similarity, as shown in Supplementary Fig. 3b; data in Supplementary Data 4. (**b**) Biological and toxicological complexity of the PTGS components defined as the proportion of results (above a set statistical threshold) in each analysis category ascribed to the component gene set. Numbers above bars denote the numbers of genes in each component. Details of the data are found in Supplementary Data 3–7. (**c**) Frequency plot of the upstream regulator enrichments for the PTGS components depicting multiple transcriptional regulators associated with stress responses, inflammation and with cell division. For data and further related analyses, see Supplementary Fig. 6b and Supplementary Data 5.

A number of different approaches were taken to validate the functionality of the PTGS (Figs 1b and 3, Supplementary Fig. 6 and Supplementary Data 3–8). The PTGS gene lists were enriched in a variety of basic biological and metabolic processes associated to growth inhibition, cellular cytotoxicity and stress response pathways, as well as to pathological effects in liver, kidney and heart; this analysis is plotted as an eye diagram with reference to highly associated instances (drug/cell line-pairs) (Fig. 3a). The pathological effects included changes typically associated with adverse drug reactions and those seen in repeated-dose toxicity studies of laboratory animals, for example, hepatic fibrosis^4^,^12^,^16^,^36^. Components A-C enriched most strongly for liver necrosis/cell death, whereas E and K enriched for liver cell proliferation, among other organ effects. The analysis also indicated enrichment for receptor-mediated toxicity, for example, Aryl Hydrocarbon Receptor Signaling and LXR/RXR Activation.

The genomic structure complexity of PTGS involved overall 1331 genes (716 up-regulated and 835 down-regulated, meaning that around 200 genes had up-regulation in one or several components, and down-regulation in others), 101 molecular networks and 97 transcriptional regulators (Supplementary Data 3). Regarding the respective components, the proportional network complexity varied extensively, and was only partially related to the gene numbers (Fig. 3b). Components towards the higher toxicity end exhibited mostly up-regulated genes, whereas the lower toxicity end components showed mostly, or even entirely, down-regulated genes (Supplementary Data 3). Component C contained many genes associated to many variables, whereas components G and B associated to many variables from fewer gene numbers. The number of upstream regulators also varied extensively among the components. Commonly involved transcriptional regulators, spanning three or more components, are shown in Fig. 3c. Totally 19 of these regulators are found in the 1331 gene set that constituted PTGS (Supplementary Data 5). Components such as G enriched for regulatory factor gene signatures (for example, TP53, NFKBIA), inflammation-related gene ontology categories as well as stress from DNA damage and reactive oxygen (Supplementary Fig. 6). Components E and K enriched for cell cycle and cell division related categories, for example, S phase of mitotic cell cycle, as well as related regulators including MYC, CDKN2A and E2F1. Of all the regulators, P53, EP300 and CDKN2A were associated with the largest numbers of components (Fig. 3c). The functional associations of the components based on gene-level analysis thus reflected the component-level clustering based on CMap instances; indicating that many aspects of the probabilistic model are preserved within the gene lists (Supplementary Fig. 3b). Comparison to the transcriptional regulators addressed in the comprehensive ToxCast project^21^ indicated coverage of 14 of the 35 regulators identified in the PTGS (Supplementary Data 5). The transcriptional regulators non-examined in ToxCast were distributed evenly across the 14 components of the PTGS, indicating potentially a different coverage of cytotoxicity mechanisms relative to the ToxCast assays; the PTGS genes overall matched 22% of the genes annotated to ToxCast, see Materials and Methods.

### PTGS-based grouping outperforms structure-based grouping

**Figure 4:**
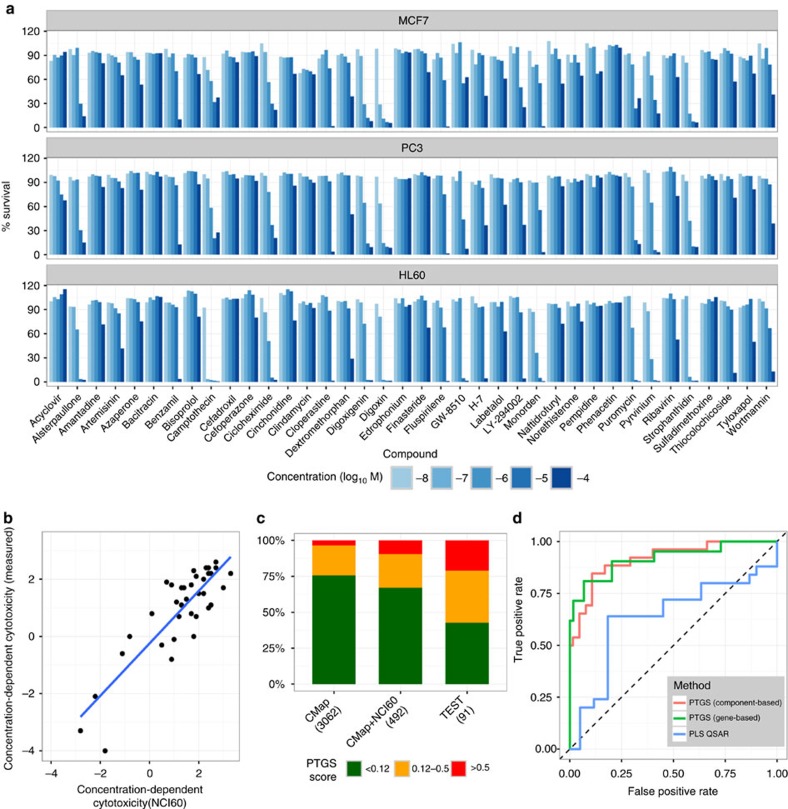
High-throughput screening cell-based validation of PTGS to predict cytotoxicity in the CMap database. (**a**) Cell survival measured in the three CMap cell lines at different concentration levels for 38 non-NCI-60 CMap compounds. (**b**) Concentration-dependent cytotoxicity values of 16 compounds (36 instances) indicated data agreement between the NCI-60-based test and the chosen cytotoxicity assay (ATP content) (Pearson correlation 0.86). As shown, the classification of toxic versus non-toxic repeated in 32 of 36 instances, and the four instances where this changed had a score close to the cut-off in both data sets. (**c**) Proportions of CMap, CMap/NCI-60 crossover and validation (test) instances predicted by the PTGS to have been measured above the GI_50_-level show a balance of toxic and non-toxic treatments (numbers tested shown). About 25% of 3062 CMap profiles are predicted to be above the GI_50_ levels. (**d**) ROC curves indicating the cytotoxicity-predictive performance of the gene-based, component-based and the Partial Least Squares QSAR methods. The AUC values were 0.92 (*n*=80), 0.91 (*n*=91) and 0.64 (*n*=85), respectively. Further details of the QSAR analysis are in Supplementary Fig. 7. For screening data see Supplementary Data 9.

The components variably enriched for particular structural and functional classes among the CMap compounds, for example, A–C were enriched for protein synthesis inhibitors and cardenolide glycosides (Fig. 3a and Supplementary Data 8). Grouping of diverse classes of compounds to specific components supported applicability of the PTGS tool as such to connectivity mapping and compound grouping. Cell culture experiments were therefore designed to challenge the fact that structure basis is so far the one accepted means of grouping compounds for avoidance for toxicity testing in regulatory contexts^22^,^24^. Cytotoxicity screening of 38 CMap test compounds, for which such data are not available in the NCI-60 data, and a set of 16 NCI-60-assessed controls demonstrated a wide range of cytotoxicity effects, and moreover verified the comparability of the chosen cytotoxicity assay relative to the NCI-60 assay (Fig. 4a–c). Both gene set-based and ‘component-based’ scores predicted the cytotoxicity of the non-NCI-60-assessed compounds with high sensitivity and specificity (Fig. 4d), and consistently outperformed predictions generated from quantitative structure-activity relationships (QSAR) analysis. This result was obtained applying either the structures for 201 of the 222 training compounds (448 of the 492 instances with cytotoxicity data), or the 35 structures of the 38 validation compounds (85 of the 91 instances) (Fig. 4, Supplementary Fig. 7 and Supplementary Data 9).

### PTGS predicts dose-dependent liver toxicity

**Figure 5:**
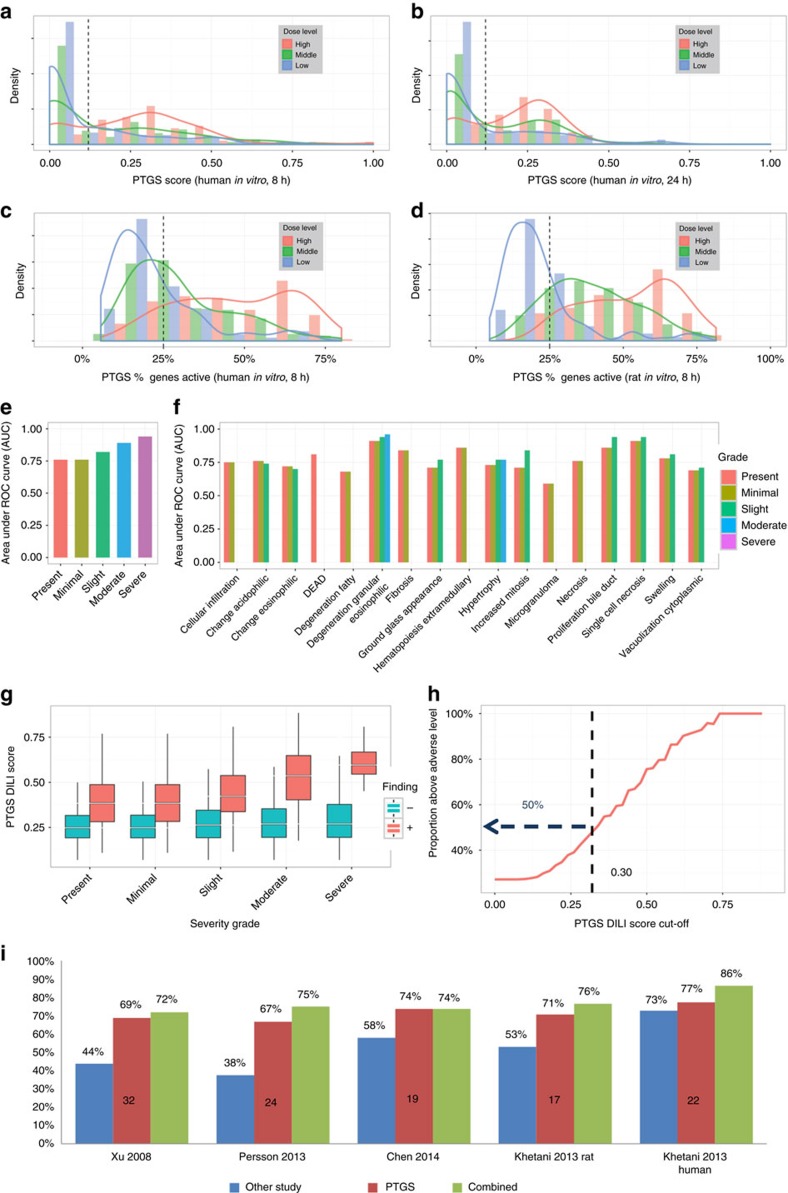
Validation of the PTGS using *in vitro* and *in vivo* profiles from the TG-GATEs toxicogenomics database. The increase with dose in the proportion of treatments exceeding the virtual GI_50_-level (dashed line) in human hepatocytes measured at (**a**) 8 h (*n*=388) and (**b**) 24 h (*n*=394) and in (**c**) human (*n*=388) or (**d**) rat (*n*=419) hepatocytes measured at 8 h, using either the component-based (**a**,**b**) or the gene-based (**c**,**d**) methods (Supplementary Data 10,11). The PTGS DILI score (for analyses see Supplementary Data 12–15, Supplementary Fig. 8), defined as the score given by the most sensitive component from among G, H, I and N, (**e**,**f**) predicts the severity grade (denoted by colour) and covers 17 different types of histopathological changes observed in repeated dose treatments of rats for up to 28 days. (**g**) Separation between positive and negative classes increases with the severity of histopathological changes from present to severe; *n*=463, 448, 282, 116 and 30 of 1689 total. (**h**) Defining a threshold for the score above which more than 50% of the observations have histopathological changes present (dashed line). (**i**) The ability of PTGS to predict clinical exposure levels raising DILI concerns was tested and compared to other *in vitro* assays. Numbers of matching compounds with rat hepatocyte data are indicated inside red bars. PTGS, by itself, outperforms the other approaches, and in combination with other hepatocellular-based assays achieved a positive predictive ability of 72–86% without a loss of specificity (further details in Supplementary Fig. 9 and Supplementary Data 16–18).

The applicability of the PTGS scoring concept was next assessed in relation to non-dividing normal hepatocytes and rat liver pathology data in the TG-GATEs toxicogenomics database (Figs 1b and 5a–h and Supplementary Data 10–15). Applying component-based scoring, human hepatocyte experiments generated increased scores with concentration at both 8 and 24 h exposures (Fig. 5a,b). Machine learning-based inference with TG-GATEs 28-day study data was then used and resulted in the selection of components G, H, I and N, as they had the highest liver toxicity predictive ability (see Materials and Methods; Supplementary Data 13–14). Capture of dose–response applying gene-based scoring with these components was verified in human and rat hepatocytes (Fig. 5c,d). Scoring using these components predicted diverse pathological changes in 45 combinations of pathological findings and severity grade, covering 1689 distinct treatments with 143 compounds in the rat liver 28-day repeated dosing data set, and thus constituted a functional DILI score (Fig. 5e,f). All 17 types of pathological effects were captured at high sensitivity and specificity, including severity grades (Fig. 5e–g; Supplementary Data 15). The endpoints included liver necrosis/cell death, ground glass appearance, fibrosis, hyperplasia/hyperproliferation (swelling), cholestasis (degeneration, fatty) and further pathologies (Fig. 5e,f), as well as liver pathologies as aggregate endpoints according to grade and even ‘death’, the latter being the one organism-level endpoint scored. These results exemplified effective PTGS-based extrapolations from cells to organ-level, as well as between species.

### PTGS broadly predicts human drug-induced liver injury

The hypothesis was thereafter tested that toxicogenomics changes measured *in vitro* can be used to predict DILI potential in human patients (Figs 1b and 5i, Supplementary Fig. 9 and Supplementary Data 16–18). For *in vitro*-based prediction we used the *in vivo* phenotype ‘presence of toxicity’ that is, the presence of pathological findings in the animal liver, to tune the predictions (see Material and Methods). A threshold for the magnitude of the score was set at a level above which at least 50% of instances showed pathological changes (Fig. 5g,h). Basis for predicting clinical exposure levels of concern is shown and explained in Supplementary Fig. 9a and b, including legend. Two withdrawn drugs (nimesulide and benzbromarone) and one drug with a good safety profile (aspirin) illustrate the calculations. Associated to idiosyncratic DILI, nimesulide and benzbromarone are metabolically converted drugs for which the mechanism of action/toxicity is not known precisely and likely to vary between patients^5^,^6^,^37^. The PTGS DILI score is activated in a dose-dependent manner by both compounds, in hepatocytes (Supplementary Fig. 9b) and rats (Supplementary Data 12). Interestingly, aspirin at the highest doses, also caused liver injury and activates the DILI score, reaffirming that dosing and exposure needs to be taken into account when assessing compound toxicities.

A literature search of the TG-GATEs data thereafter enabled annotating 68 compounds with their therapeutic C_max_ values and results on liver toxicity. The gathered information implied that the data set in several instances reflected therapeutic doses also below the C_max_ values (Supplementary Data 16). The PTGS DILI score was then applied to derive a safety margin of exposure relative to the therapeutic C_max_ concentration (see Materials and Methods). The DILI potential of the annotated agents was found to be predictable to a level of 100% specificity and 71% sensitivity with rat hepatocyte data (Supplementary Fig. 9c and Supplementary Data 17). Differently, the similar analysis with human hepatocytes indicated 100% specificity and 58% sensitivity. Interestingly, while the rat hepatocyte data performed best overall, perhaps owing to low sample variation, the human data performed better and exceeded the rat performance, by 73% versus 71%, in predicting the most clinically troubling withdrawn and boxed warning labelled toxicities in the Liver Toxicity Knowledge Base^32^. The analyses provided the similar level of prediction with the subset of drugs labelled ‘most DILI-concerning’. Compared to other *in vitro* methods applied to predict DILI^38^,^39^,^40^,^41^, PTGS provided better predictive performance, and moreover, provided further improved prediction levels in combination with the methods (Fig. 5i, Supplementary Data 18).

## Discussion

This study represents a large-scale data analysis aimed at addressing broadly human health and safety of chemical compounds, including drug molecules. Coupling of omics data to the prediction of dose-dependent induction of cytotoxicity effects resulted in the first ever description of a PTGS. Representing a comprehensively validated construction, it captures a wide range of dose-dependent cytotoxicity effects, and therefore serves to improve prediction of hepatocellular toxicity and liver pathologies in humans and rats relative to existing methods.

The data fusion underlying the PTGS tool involved extensive probabilistic modelling-driven transformation, compacting and selection of the data points, instances, compounds, components and genes (summarized for overview in Table 1). The level of reduction was to between 1 and 10% of the input data; for example, the CMap was reduced, transformed and decomposed to 0.7% of the original data size, and altogether, 22% of the gene expression alterations, that is, 1331 versus 6064 genes (11% of all measured transcripts), connected to cytotoxicity-related transcriptomic changes. As around 25% of the CMap gene expression profiles likely reflect cytotoxicity above GI_50_ (cf. Figs 2c and 4c), the PTGS is based on, and covers, a significant portion of the CMap gene and sample dimensionality. Giving further support to this assumption, the 14 components included those of the overall 100 original components with the most extensive gene expression changes (cf. Fig. 2c). The PTGS calculation methods are most likely equally applicable to both microarray and RNA-seq gene expression data. Because of the ability of RNA-seq to detect alterations more sensitively than microarrays, it may detect activation of PTGS at lower doses, an issue that would be testable in sufficiently large and matching data sets. Further studies could also consider the PTGS approach and scoring concept using proteomics and metabolomics data.

Overall, the described ‘big data-driven’ analysis enabled: (1) a virtual cellular cytotoxicity probability estimate intrinsic to omics-data, (2) calculation of toxic exposure thresholds for compound effects, (3) grouping of compounds into mechanistically similar classes, (4) assessment of the cytotoxicity of CMap profiles, with implications for using the database and gene expression profiles generally for mode of action studies, (5) coverage of adverse outcome-coupled toxicity effects involving a multitude of transcriptional regulators, (6) prediction of known measured liver toxicity and pathology effects in the TG-GATEs, including a ‘severity-grade response’, from data obtained in cultured cells (for example, rat/human hepatocytes) and laboratory animals (for example, in rats) and, finally, (7) prediction of exposure levels raising concern for human DILI from hepatocyte experiments. The latter analysis includes opportunity for improved preclinical *ab initio* prediction of safety margin for novel drug molecules, while serving in a complementary manner to raise the prediction level of existing evaluation tests (range 14–38%; cf. Fig. 5i), including a commercially available test. An *ab initio* testing of a previously non-tested compound under the PTGS concept would generate a probability score for both cytotoxicity and liver pathology. Under a qualified, preclinical efficacy drug testing protocol, a range of human-relevant concentrations would be derived that could be assessed with PTGS to then include risk-prediction of DILI to this analysis. The overall results would constitute a qualitative and quantitative hepatotoxicity/DILI measure, including coverage of mild to overt effects. The DILI prediction scoring could likely be further refined from standardizing drug concentrations relative to the therapeutic C_max_ more precisely and by incorporating further negative control compounds. Furthermore, future connectivity mapping-based testing with PTGS components to predict *in vivo* outcomes from *in vitro* hepatocyte toxicogenomics data would likely indicate further the applicability of PTGS in relation to specific pathological states. Applying the concept to capture further organ toxicities is an even further interesting task, agreeing with that the bioinformatics assessment indicated component association to a diversity of heart and kidney conditions (cf. Fig. 3a; Supplementary Data 4).

Being the focus of the current study, DILI is multifactorial, sometimes receptor-mediated or occurs in response to gross stress^5^,^6^. Idiosyncratic DILI occurs unpredictably, with variable length latency and sometimes without dose-dependency^5^,^6^,^32^,^33^. Interestingly, the PTGS classified idiosyncratic DILI-drugs in dose-dependent manners, for example, nimesulide (cf. Supplementary Fig. 9b). The predictive components (G,H,N,I) might therefore quantitatively evaluate a relatively broader complexity of DILI-inducing mechanisms than existing tests. Interestingly, these components associated to lower cytotoxicity in the CMap training data set (cf. Fig. 2c, Supplementary Data 3), implicating that relatively milder, rather than severe, cellular toxicity effects might better reflect at least certain DILI mechanisms. We hypothesize overall that the current work could serve to stimulate the integration of component models in future DILI studies, and generally, scoring concepts into AOP-based risk assessment strategies. For example, the PTGS component gene sets are enriched in liver fibrosis-related gene signatures and detect hepatocellular damage markers in the fibrotic mechanism. Thus, PTGS could be used to ‘biomark’ key events detailed in the corresponding AOP^20^,^42^ (cf. Fig. 3a and Supplementary Fig. 6). Such mapping of the PTGS would then constitute a hybrid data and knowledge-driven approach for novel AOP developments.

Capturing potentially the multitude of gene activities that underlie the dose-dependency of many cytotoxicity mechanisms within a reduced feature set, the PTGS-generating approach can be considered as a model for defining toxome descriptions^43^. The analyses overall applied 84 × 10^6^ data points and 1217 compounds to generate the PTGS (cf. Table 1), and assessed 250 × 10^6^ data points overall, including the TG-GATEs data. Variably from 140 to 170 compounds were assessed to validate the scoring concept. Being a small but important part of the current study, the toxicogenomics-based scoring outperformed the QSAR-based toxicity predictions (cf. Fig. 4d). Regulatory agencies such as the European Chemicals Agency and the United States Environmental Protection Agency are increasingly advocating for the inclusion of trancriptomics data and new approach methodologies in chemicals risk evaluation^1^,^2^,^11^,^18^,^24^. Thus, the demonstration of this mostly expected outcome fills the important role of implying broad applicability of the PTGS concept also outside of drug discovery studies. Challenging to traditional means of optimizing biological testing practices and coupled mechanistic reasoning, the CMap-derived PTGS establishes that even tumour-derived cellular models with known aberrant metabolism and differentiation capacity can be used to capture mechanisms that predict *in vivo* dose-dependent liver toxicity in a cross-organism manner. The rich variety of agents assessed in the CMap, including direct acting cytotoxic cancer drugs, may potentially underlie the capturing of cytotoxicity/pathology of agents requiring metabolism to exert their effects. Although complex in overall structure and function, PTGS is naturally suited for analysis in high-throughput transcriptomics assays, for example, the Tox21 platform^10^,^23^. We emphasize finally the full adherence of our study and the PTGS concept to replacing animal testing protocols with quantitative systems toxicology and human cell culture-based experiments, arguing overall for broad and opportune applicability of the PTGS concept in diverse future safety testing practices.

## Methods

### Pre-processing of the connectivity map data set

To decrease the low-intensity noise in the data the Connectivity Map (CMap) raw data^25^, the CEL-files (downloaded from http://www.broadinstitute.org/cmap/; and E-GEOD-5258 for build01) were robust multi-array normalized with R/Bioconductor-package aroma.affymetrix and mapped to Ensembl gene identifiers (custom CDF version 12, http://brainarray.mbni.med.umich.edu/Brainarray/Database/CustomCDF/CDF_download.asp)^44^,^45^,^46^,^47^. Results from the most abundant microarray platform (HT-HG-U133A) were used, containing measurements for the three cell lines MCF7, PC3 and HL60. To further reduce the noise in the expression data the 5% of the genes displaying the highest variance in the control measurements were removed^48^. Differential expression was then computed as the log_2_ ratio between the drug treatments and respective control measurements. The CMap measurements had been made in batches. In the case of multiple negative controls per batch, adapting established procedures, a more robust control was formed by calculating a mean of the control measurements after first removing, as an outlier, the control with the highest (Euclidean) distance to the other controls. To balance between the varying sample sizes for different compounds, the instance for each compound and cell line with the strongest effect, measured as the highest (Euclidean norm of) response, was selected for further analysis. A total of 18 compounds in the data set had more than one (and mostly two) concentrations. To balance between the varying sample sizes for different compounds, the instance for each compound and cell line with the strongest effect, measured as the highest (Euclidean norm of) response, was selected for further analysis. The resulting gene expression data consisted of 3062 treatment instances (compound and cell line pair) and profiles for 1217 distinct compounds in the three cell lines (MCF7, PC3 and HL60, with 1203, 1131 and 728 instances per cell lines, respectively). For further details see Table 1.

### Probabilistic component modelling

It was assumed that compound treatments may activate multiple response patterns, each of which may be shared by several compounds^49^. These patterns were identified with probabilistic modelling that decomposes the chemical-induced transcriptional variation into components of interrelated activity. Biological prior knowledge was brought into the analysis while also reducing the data dimensionality with Gene Set Enrichment Analysis (GSEA)^50^. GSEA was computed (Java software version 2–2.05, http://www.broadinstitute.org/gsea) using 1321 distinct C2-curated gene sets v2.5 from the Molecular Signature Database (http://www.broadinstitute.org/gsea/msigdb). The false discovery rate q value (FDRq), which GSEA produces to represent the strength and direction of the gene set activation, was quantized to non-negative integer values with the transformation min(round(−log_*2*_FDRq))–1, 0), separately for the positively and negatively activated genes in the gene sets, resulting in activation counts for 3062 instances over 2642 gene sets.

The latent Dirichlet allocation^34^,^35^ (LDA) model was then used to identify transcriptional response patterns from the gene set activation count data. Each resulting component associates probabilistically a subset of the treatments with a subset of the gene sets. Each component thus represents a specific chemical-induced response pattern, interpretable based on the associated gene sets. To select the number of components, an external validation set describing the functional similarity of the drugs based on their known protein targets and ATC (Anatomical Therapeutic Chemical, http://www.whocc.no/atc_ddd_index/) codes was used^48^. Drug target information was obtained from ChEMBL (https://www.ebi.ac.uk/chembl/), DrugBank (http://www.drugbank.ca/), DUD (http://dud.docking.org/) and ZINC (http://zinc.docking.org/). In addition targets and ATC codes for the CMap compounds were extracted from publicly available sources^26^. Drugs sharing fourth-level ATC codes were treated as functionally similar for the purposes of this analysis. In total, 4427 associations between 821 CMap compounds and 796 targets or ATC codes were used. The component count which maximized the performance in retrieving (that is, predicting) drugs sharing these annotations was chosen from the set of 20, 50, 100, 150 and 200. The posterior distribution of the model parameters was computed with collapsed Gibbs sampling. For the hyperparameters controlling the sparsity of the model, gamma hyperpriors were applied with fixed parameters and their posterior was estimated with Metropolis sampling.

### Toxicological profiles from NCI-60

Toxicological profile data were downloaded from the NCI-60 DTP human tumour cell line screen web site (http://dtp.nci.nih.gov/docs/ cancer/cancer_data.html)^30^. The data set has three reported drug response values: GI_50_ (50% growth inhibition), TGI and LC_50_ (50% lethal concentration) for 59 different cell lines. These values have been inferred from measurements covering typically five concentration values, most common range being from 10 nM to 100 μM (or from −8 to −4 log_10_ *M*). The NCI-60 and CMap instances were matched based on the compound names. In addition, alvespimycin and tanespimycin, named 17-DMAG and 17-AAG in NCI-60, respectively, were added manually. The three drug response values were extracted from NCI-60 data for in total 222 CMap compounds and 492 cross-over measurement instances on the three cell lines (MCF7, PC3 and HL60; with 197, 179 and 116 instances per cell line, respectively), averaging over multiple measurements when available. The resulting NCI-60 data are provided in Supplementary Data 1.

### Concentration-dependent cytotoxicity

Concentration-dependent cytotoxicity was defined as the difference of the logarithmic CMap concentration and GI_50_ values, that is, log_10_(CMap concentration)−log_10_(GI_50_). Cellular growth inhibition above the GI_50_-level was used as a cut-off to classify the 492 cross-over instances as either cytotoxic (*n*=121) or non-cytotoxic (*n*=371), as shown in Fig. 2.

### Defining the Predictive Toxicogenomics Space (PTGS)

As the CMap generally includes one concentration assessment (10 μM), dose-dependent cytotoxicity was modelled across compounds under the *a-priori* assumption that compound-induced transcriptomic responses are subject to the compounds’ intrinsic potency to cause cytotoxicity (for additional details see Supplementary Fig. 2). The 100 components produced by the probabilistic model covered the full space of transcriptional responses caused by the 3062 CMap measurement instances. Associations of the components to cytotoxicity were sought by evaluating their ability to predict the concentration-dependent cytotoxicity for the cross-over instances. The concentration-dependent cytotoxicity values have the highest density around GI_50_, making the data set ideal for predicting relatively low levels of cytotoxicity. Thus, a classification model was trained to identify whether an instance had been measured above the GI_50_-level. The 100 LDA-components were first ranked based on their probability-weighted mean concentration-dependent cytotoxicity values over the 492 training instances. The mean cytotoxicity values were computed as


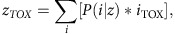


where *i*_TOX_ is the concentration-dependent cytotoxicity in relation to GI50 and where the normalized probabilities *p*_*n*_*(i|z)* for the training instances *i* to belong to component *z* were computed as


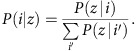


Starting with components with the highest associated cytotoxicity and using the sum of the component probabilities to calculate a predictive score, the 100 components were progressively included in the model. The cumulative concentration-dependent cytotoxicity classification performance over the test instances was evaluated, providing area under the ROC curve values (AUC) for each component count (Fig. 2b). A perfect model would have an AUC-ROC of 1 and an AUC-ROC of 0.5 indicates a random classifier. To focus on the components with the highest relevance to cytotoxicity, the number of components was chosen where the AUC value reached 95% of the highest value, resulting in a trade-off between interpretability and the highest predictive performance. Cytotoxicity-predictive performance is expected to decline with a large number of components, as non-relevant components are included, see Fig. 2a,c. The resulting top 14 components were chosen to define the Predictive Toxicogenomics Space (PTGS). The components were labelled from A-N, with component A having the highest probability-weighted mean concentration-dependent cytotoxicity value. The probability of an instance to belong to the PTGS components, calculated as the sum of their individual contributions, was thereafter used as a predictive score for its cytotoxicity.

### PTGS associated genes and a gene-based scoring method

The PTGS and each of the 14 components were then characterized further by a most active set of instances and set of genes. The most active genes were obtained for each component as follows: top instances having the largest *P(i|z)* were chosen, thresholding at cumulative probability reaching 0.2. The same was done for the gene sets. The differential expression of all genes included in the top gene sets was evaluated within the respective top instances with a standard two-sided t-test. A set of PTGS associated genes was defined based on *t*-test *P*-value cut-off 0.01, after Bonferroni correction for multiple testing (labelled ‘PTGS Core’). This subset of 199 genes strongly associated to the PTGS in general, but was not further sub-divided by component membership. To generate a component-specific list, and considering that Bonferroni correction would be too conservative, a ranked list of genes thresholded at the 0.01 level was derived, with the rationale that the higher a gene is on the list, the more evidence there is for it being informative in characterizing the component. The 14 gene lists are referred to as ‘PTGS-associated genes’ and listed in Supplementary Data 2. Thus a total of 1331 most active genes, as indicated by the *P*-values, characterized the individual components. They were used for functional enrichment analysis and as cytotoxicity-predictive genes/features.

To further simplify PTGS scoring, a gene-based scoring method using gene set enrichment analysis was implemented. The ROAST test within the limma R/Bioconductor package^51^,^52^ was selected as it has advantageous characteristics, for example, in relation to robustness to sample heterogeneity^53^. It also uses the variance-modelling strategies implemented in limma to improve performance with small sample sizes^52^. The score was calculated using non-directional (that is, mixed) *P*-values and the ‘floormean’ summarization. This method detects instance-cytotoxicity when as few as 25% of genes in the set are differentially expressed, defined as |*z*|>sqrt(2)^51^, with *z* denoting *z*-score based on limma differential expression analysis. Thus the gene-based PTGS components are defined as a combination of the gene sets and a gene set testing statistic, that is, method used to derive the score from them.

To test the gene-based scoring method, instances corresponding to the 222 NCI-60 DTP and CMap crossover compounds within batches with at least 3 replicate untreated control measurements were analysed. Scores from replicated treatments were averaged to obtain 482 unique instances in the three CMap cell lines (MCF7, PC3 and HL60; with 176, 156 and 150 instances per cell line, respectively). A virtual GI_50_ estimation using the PTGS-associated genes is thus calculated as follows: (1) Normalize data to remove systematic variation. (2) Fit treatments and controls to a linear model using the R/Bioconductor limma/eBayes method^52^. (3) Calculate activities of the PTGS-component derived gene sets (A-N) and the PTGS_ALL gene set (which contains all of the 1331 PTGS-associated genes). (4) Use results from PTGS_ALL to predict GI_50_-level of activation (utilizing *q*<0.05 and proportion of active genes >25% as thresholds).

### Characterization of the PTGS by enrichment analyses

Biological interpretations of the PTGS were enabled by the enrichment analysis of the component gene sets using Ingenuity Pathway Analysis (IPA, application version 220217, content version 16542223; build: 430520M and 31813880 content version for networks) and Gene Ontology (GO) enrichment analysis (R package topGO, version 2.12.0)^54^,^55^. The results were visualized with eye diagrams^56^. For visual interpretation, the results were thresholded at *P*-value 0.001, and at least three genes were required to be annotated to each GO category, IPA toxList or IPA regulator. IPA upstream regulator analysis results were further filtered to include all regulators that were enriched both in the overall gene set of 199 genes (PTGS Core) as well as in any of the components, and additionally connected to other regulators via a mechanistic network^55^ to give further evidence of a genuine regulatory relationship. Furthermore, since the core set did not cover all biological functions, highly overrepresented regulators (*P*-value <10^−5^) that occurred in at least one third of the 14 components were added to the eye diagram. To compare upstream regulator analysis results with the ToxCast assay information^21^, information on genes associated with the assays was downloaded (http://actor.epa.gov/actor/faces/ToxCastDB/GenesAssocAssays.jsp) and matched with Ingenuity upstream regulators on the basis of the gene symbol. Association of cytotoxicity to biological complexity was calculated for each type of analysis by, 


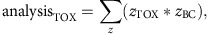


where *z*_BC_ describes the biological complexity of a components, computed as *n*_results(Zi)_/n_results(Z)_, where as *Zi* denotes each individual component and *Z* all components and *n*_results_ is the number of statistically significant results produced by an analysis, as detailed in Supplementary Data 3.

### Hierarchical clustering and principal components analyses

Grouping of the components was assessed by hierarchical clustering of CMap instances mapped to the PTGS components with ggdendro (v. 0.1–20), using default settings. Furthermore, Principal Components Analysis (PCA) with the made4 (v. 1.44.0)^57^ R package and visualization with the scatterplot3d (v. 0.3–37) was used to assess overall grouping. Clustering of the gene sets was also investigated with PCA, treating presence of absence of genes within a component as a Boolean vector.

### Cell culture and *in vitro* cytotoxicity predictions

To validate the predictive performance of the PTGS, a set of CMap instances that were not included in the NCI-60 data set were assessed for cytotoxicity (Fig. 4a). CMap cell lines MCF7 (ATCC HTB22), PC-3 (ATCC CRL-1435) and HL-60 (ATCC CCL-240) were obtained directly from American Type Culture Collection (LGC Promochem AB) and maintained at 37 °C with 5% CO_2_ in a humidified incubator according to provider’s instructions. As described previously, the cell lines were grown in larger volume to make assay ready cells, tested for mycoplasma using PCR-based test kit and frozen in several ampules^58^. Before screening, the cell number was titrated to ensure that cell proliferation remained in a linear-exponential phase throughout the experiment (1,000–2,000 cells per well were plated)^59^. Each experiment was performed from unique assay ready cells (same passage). Data quality and assay comparability were first verified by replicating the measurements for 36 instances for 16 different compounds already measured in NCI-60. For the controls, using the formula *N*=*((Zα+Zβ)/C)*^2^+3, adequate sample size (>30) were chosen to be able to detect with a power (1−*β*) of 0.8 a correlation coefficient (*r*) of 0.5 or greater with 0.05 two-tailed alpha-level (*α*, Type I error rate); *Z* is the *α* or *β z*-score and *C*=0.5 × ln((1+*r*)/(1−*r*))^60^. The test assumes normality. In total 91 instances for 38 unique compounds were then chosen for measurement according to pre-established criteria (Supplementary Data 9): instances from the very top of the list (highest expected cytotoxicity) as well as instances with very low score (controls with expected low cytotoxicity) were included. Compounds were purchased from Sigma-Aldrich and dissolved in DMSO. To reduce plate-level signal bias, compounds were distributed randomly on the 384-well plates and diluted from a single master plate each time. These 91 instances were then measured using CellTiter-Glo Luminescent Cell Viability Assay (Promega) on cells treated with the compounds at five concentrations spanning a 10,000-fold range for 72 h in 384-well plates using automated methods to reduce investigator bias. The raw concentration response data were processed, as explained in the NCI-60 web page^30^ (http://dtp.nci.nih.gov/), and GI_50_ values were computed using vehicle (DMSO)-only treated cells cultured in the plates for 72 h (corresponding to 0% GI) and for 0 h (corresponding to starting cell number, TGI). Predictive ability (retrieval) versus the measured GI_50_-level was tested with area under the ROC curve analysis; in addition to chemical structure-based analysis (see below), both component-based and gene-based PTGS analyses were carried out. The component-based analysis encompassed 91 instances, whereas the gene-based analysis had data on 80 of these instances. The R-package pROC (Version 1.7.2) was used for statistical analyses related to ROC curves^61^. Data is available at FigShare (10.6084/m9.figshare.4954583).

### QSAR analysis

PTGS was compared with predictive models based on the chemical structures of the compounds. Various QSAR approaches^62^ were tried, including partial least squares^63^ (PLS), decision trees and supervised Kohonen maps^64^. PLS models were found to perform equally well or better than decision trees and Kohonen maps, and only those details are reported. No support was found for the presence of non-linear patterns. The training set was defined by chemical structures from the NCI-60 data set. For a few compounds it was not possible to confidently map the chemical name to a structure, resulting in structures for 201 of the 222 compounds (448 of the 492 instances). The validation set was based on the experimental validation data and, based on theoretical descriptors^65^ and molecular signatures, 35 chemical structure representations of the 38 compounds were formed (85 of the 91 instances). These descriptors were calculated with the Chemistry Development Kit^66^ R-package rcdk, version 3.1.21). This resulted in 185 descriptors and 2400 signatures with non-zero variation within the test and training sets.

PLS models were trained for the NCI-60 data set correlating the compound structures with their cytotoxicity using PLS. While compounds were classified based on the concentration-dependent cytotoxicity for PTGS, the QSAR models were built to correlate the chemical structure with their GI_50_ values. Following previous studies, a −5 log_10_ *M* cut-off was used, below which compounds were classified as toxic^67^. This difference is justified because the concentration-dependent cytotoxicity and GI_50_ values are highly correlated in this data set, as is also clear from the small differences between the class labels (Supplementary Fig. 2b; Supplementary Data 9). Cross validation was used to estimate the suitable number of latent variables for the final PLS models: the smallest number of latent variables was selected that gave performance within one s.d. of the highest mean performance. The regression models were then used to predict the cytotoxicity classes of the test set compounds (toxic or non-toxic). This performance in the test set, as measured by ROC curves, was compared with PTGS component predictions, and y-randomization models were used to establish a baseline. To ensure conformity between the complete and reduced data sets (85 versus 91 instances), the performance of the component-based PTGS approach was additionally evaluated in exactly the same setup in which the QSAR was run, resulting in an AUC value equal to the reported PTGS performance. Thereafter, Tanimoto similarity measurements between the compounds were made to evaluate whether the diversity between the compounds in the data sets could explain the performance of the PLS.

### Open TG-GATEs data normalization and pre-processing

Liver-related treatments from the Open TG-GATEs^16^ database were employed to assess the predictive ability of the PTGS-associated gene sets against external data. The complete data was downloaded from the publisher’s web site (http://dbarchive.biosciencedbc.jp/en/open-tggates/download.html) and custom processed, unless otherwise stated. Raw data is also available at ArrayExpress (E-MTAB-800, E-MTAB-799, E-MTAB-798 and E-MTAB-797) and through the EBI Dixa data warehouse (http://wwwdev.ebi.ac.uk/fg/dixa/index.html): diXa-005, DIXA-006 and DIXA-008. As detailed by the creators, the data assayed 143 compounds on 6765 genome-wide microarrays and 1689 treatment instances from repeated dose treatments of Sprague–Dawley rats, employing three dose levels, that is, low, medium and high in the 1:3:10 ratios with time-matched controls^16^. To generate the profiles, organs had been obtained from the animals 24 h after the last dose of repeated administration for 3, 7, 14 and 28 days with 3 animals in each treatment group. Two types of *in vitro* study, primary hepatocytes from Sprague–Dawley rats (3370 Affymetrix microarrays; 1255 comparisons/instances) and from human donors (2605 Affymetrix microarrays; 941 instances), were also used. Hepatocytes had been treated with three dose levels that is, low, medium, high with 1:5:25 ratios utilizing time-matched controls, and measured with gene expression analysis 2, 8 and 24 h after treatment. To normalize the data, the robust multi-array method was employed with the R/Bioconductor package simpleaffy (v. 2.40.0) using mappings of Affymetrix probes to Ensembl gene identifiers from custom cdf files, using the hgu133plus2hsensgcdf version 17.1.0 for human and the rat2302rnensgcdf version 19.0.0 (refs 44, 45, 46, 47). Separately processed Open TG-GATEs data were employed to validate the component-based analysis. Rat hepatocyte and liver gene expression profiles (CAMDA 2013; http://dokuwiki.bioinf.jku.at/doku.php) were downloaded as FARMS-normalized pre-processed data (log_2_ fold change relative to respective control treatments), with replicates collapsed to a single treatment instance. Uninformative genes according to the FARMS metric (0.1 threshold) were filtered out of the data set^68^. In total, data for 131 compounds in rat hepatocytes (1177 instances) and rat livers (1568 instances) was obtained for this analysis.

To obtain pathological severity scores for each unique treatment instance, data on pathological findings was downloaded (http://dbarchive.biosciencedbc.jp/en/open-tggates/download.html) and processed into table format using R workflows and packages tidyr, reshape2 and dplyr^69^. Typically each treatment included 6 animals that were assessed for histopathological changes, while 3 of those were profiled with arrays. All findings were processed and later selected for analysis based on sample number. The type of pathological change (for example, fibrosis) and its severity grade were combined, and are here defined as endpoints. Pathology endpoints were cumulatively summed, in the order from the lowest grade-level indicated, that is, present (present+minimal+slight+moderate+severe), minimal (minimal+slight+moderate+severe), slight (slight+moderate+severe), moderate (moderate+severe) and severe (only severe samples included). Findings were also summarized, as above, on their severity grade alone. To reduce multiple testing burdens and to aid interpretation, a weighted approach producing a single score per finding was used as an alternative scoring metric throughout: pathology score=1*present+2*minimal+3*slight+4*moderate+5*severe. The endpoints were filtered to include only those with at least 10 instances, as power calculations performed with MedCalc (v. 16.8) indicated the need for >10 out of 1689 samples for significant detection (AUC > 0.75, power 0.8, type I error rate (two-tailed alpha) of 0.05). Numbers of differentially expressed genes were included for reference using *P*<0.01, absolute log_2_ fold change>0.25; multiple testing correction was done with a nested structSSI-method (Structured Simultaneous and Selective Inference for Grouped or Hierarchically Structured Data), treating all comparisons within a single compound treatment-set as a grouping variable^70^. For the tabulated pathological scores covering all the analyses, see Supplementary Data 12.

### Component selection for analysis of liver pathology

To identify and study the components most central to liver toxicity and to demonstrate the applicability of the PTGS component-based method to assess risk of agent-induced (for example, chemical compounds, drugs) liver toxicity, predictive modelling was undertaken. To begin, PTGS model-derived components were computed as in the “Defining the Predictive Toxicogenomics Space (PTGS)” section: Broad Institute GSEA tool was run on differential expression (fold-change-based) results using the R/Bioconductor limma version 3.20.9 and, as for the CMap data, the output was quantized. To update older symbols, the gene symbols were mapped to Ensembl gene identifiers for human and rat, using the multi-symbol checker tool (http://www.genenames.org/cgi-bin/symbol_checker). Based on the estimated component distributions, the individual component probabilities and the PTGS scores were computed and used for toxicity prediction. For an example, see code at Zenodo (DOI:10.5281/zenodo.570115).

Subsequently, in order to study which individual components are predictive of liver pathologies, 24 elastic net regularized regression models, one for each finding (19) and for each severity grade (5), were fitted with the 14 component probabilities as input (X-variables) and the dichotomized pathological findings as the output (y-variable); and trained using repeated (10 times) three-fold cross-validations^71^. The weighted scoring of pathological findings was employed. The findings were then dichotomized using a score at least 3 for present and minimal grades, at least 2 for the other three and at least 3 for the severity grade-weighted scores (range for N: 17-444). For robust results, only findings with more than 15 positive instances were included in the analysis. Receiver operator curves (ROC) were computed for each model using the pROC version 1.7.2 (ref. 61). Significance for the AUCs for the classifiers was estimated using two-tailed univariate Wilcoxon rank-sum statistics in R between the effected and non-effected groups. For comparison, a standard error estimate of the AUC using parametric methods is included. For the calculation of component-wise *P*-values, selective inference was carried out using the lasso penalized score test, termed lassoscore, employing the lambda values derived earlier using cross-validation^72^,^73^. Nested multiple testing procedures from the R package structSSI were used^70^, employing the adaptive Group Benjamini-Hochberg Procedure with the ‘tst’ (two step test) method and model identity, that is, the pathological finding endpoint/grade as the nesting variable or group index. A *q*<0.05 for both component and over-all model significance was used as a dual threshold. Based on these analyses, components were selected for scoring hepatic injury (Supplementary Data 3 and 13).

### Analysis of liver pathology using the gene-based method

To test the predictive ability of the component-based and the gene-based methods the rat repeated dose study was analysed using either all components or the ones which were selected earlier as being the most liver pathology predictive (that is, G, H, I and N). Gene set activities and *P*-values were computed with the ROAST method using 9999 rotations and the ‘floormean’ gene set summary statistic. In addition to the full PTGS, drug-induced liver injury (DILI) predictive scores were defined as:

Component-based DILI score=sum(prob_G_, prob_H_, prob_I_, prob_N_)

Gene-based DILI score=max(%act_G_, %act_H_, %act_I_, %act_N_)

Gene-based DILI *P*-value=min(*P*_G_, *P*_H_, *P*_I_, *P*_N_)

Where the prob-prefix refers to the component probability i.e. *P(i|z)*. As per the ROAST function, the %act is the percentage of genes which are at least marginally differentially expressed at *|z|>*sqrt(2) where as *z* denotes a *z*-score according to limma analysis and *P*.

To evaluate the gene-based scores with AUC analysis, the proportion of active genes was used for scoring. The findings were dichotomized using a score at least three for present and minimal grades and at least two for the other three; only findings with at least 10 positive instances were included in the analysis (range for *n*: 16–463 for gene-based and 11–444 for component-based). Significance for the AUCs was computed using two-tailed univariate Wilcoxon rank-sum statistics in R between the effected and non-effected groups and multiple testing corrected using the Benjamini–Hochberg procedure. Analyses using these parameters were also performed for the component-based PTGS and DILI scores. Results for component-based and gene-based analyses are tabulated in Supplementary Data 14 and 15, respectively. To further characterize the performance of the scores, for each endpoint an optimal score cut-off was computed using default settings in pROC and sensitivity, specificity and accuracy at that point was tabulated. Gene-based predictions were further characterized by parametric and nonparametric summary statistics, by identifying the proportions of outliers, by normality assumptions tests and by testing for the homogeneity of variance between effected and non-effected groups. For selected endpoints the relationship of the scores to the pathological findings was visualized with boxplots and with a cumulative distribution plot (Fig. 5). On the basis of the significance levels in predicting rat liver histopathology, the gene-based DILI scoring approach was chosen. To establish a threshold for the DILI score, scoring thresholds were plotted against the proportion of findings with histopathological changes (*n*=1689 overall and *n*=463 for the ‘present’ endpoint), and the 50% level (about two-fold enrichment of findings) was used as the decision threshold (score >0.3) in parallel with the significance level of *q*<0.05.

### Predicting human drug-induced liver injury

Human and rat hepatocyte data from the Open TG-GATEs database was analysed in combination with C_max_ values (maximal total blood concentration) from literature to predict clinical exposure levels of concern for DILI^32^,^38^,^39^,^40^,^41^,^74^,^75^,^76^,^77^ (tabulated in Supplementary Data 16). Withdrawn drugs and other labelling associated with drug-induced liver injury concern were also obtained from the Liver Toxicity Knowledge Base^33^. Assay concentrations were compared to the C_max_ values to derive a safety margin relative to the Lowest Observable Effect Level (LOEL). The approach is similar to high-content screening based studies that have been used to predict DILI from *in vitro* data, that is, omics data processed into PTGS scores is used as a high-content endpoint^31^,^32^. Safety margin was thus defined as: log10(concentration of chemical in rat hepatocytes when PTGS becomes active)–log10(human blood therapeutic C_max_ concentration). To derive a threshold for predicting DILI, negative control compounds were analysed to establish first whether the PTGS could be used to achieve 100% (or nearly) true negative rate (that is, specificity) using an acceptable safety margin of 10–100 fold above the C_max_, and subsequently to establish a threshold for safety margin with 100% specificity. The human hepatocyte data permitted the analysis of 11 negative controls and 54 compounds annotated as DILI positive, whereas the rat had 9 negative control and 55 DILI positive compounds. Compounds with a safety margin below the threshold of the negative controls were predicted as DILI positive. Comparative and combinatorial analyses in relation to representative *in vitro* methods were done using conditional array formulae in Excel. A positive result was achieved if either of the methods gave a positive DILI prediction with the shared compounds, with steps illustrated in Supplementary Data 17,18.

### Statistical and bioinformatics analyses

Nonparametric statistics were extensively used for between-group comparisons^78^, and for sample numbers below 5 variance-adjusted parametric tests were used^52^. Analyses were performed using the R statistical programming language, v. 2.15.3–3.2.3 (http://www.r-project.org/). Various R packages were used for data pre-processing and transformations^69^: tidyr (v. 0.4.1), stats::reshape (R 2.15.3–3.2.3), plyr (v. 1.8.4), dplyr (v. 0.4.3), magrittr (v. 1.5), reshape (v. 0.8.5) and reshape2 (v. 1.4.1). Statistics analysis utilized R base functions, stats/stats4 (R 2.15.3 - 3.2.3), MASS (v. 7.3–45), aod (v. 1.3), structSSI (v. 1.1.1)^70^, vcd (v. 1.4–1), glmnet (v. 2.0–5)^71^, q value (v. 2.2.2), lassoscore (v. 0.6)^72^,^73^, caret (v. 6.0–7.0), ISLR (v. 1.0) made4 (v. 1.44.0)^57^ and pROC (v. 1.7.2–1.8)^61^, foreach (v. 1.4.3) and BiocParallel (v. 1.4.3) libraries, as well as the MedCalc (v. 16.8) software. Figures were produced with the ggplot2 (v. 2.1.0)^79^, scales (v. 0.4.0), RColorBrewer (v. 1.1–2), ggdendro (v. 0.1–20), ggrepel (v. 0.5), grid (R 2.15.3–3.2.3) and gridExtra (v. 2.2.1). EyeDiagrams were produced with the custom software (2011–2012)^56^. R/Bioconductor^47^ packages were utilized for bioinformatics analyses: Biobase (v. 2.30.0), BiocGenerics (v. 0.16.1), aroma.affymetrix (v. 1.2.0)^46^, limma (v. 3.26.9)^51^,^52^, simpleaffy (v. 2.46.0), affy (v. 1.48.0), topGO (v. 2.12.0)^54^ and GO.db (v. 2.9.0). Microsoft Excel (various versions) was used for browsing and editing of tables.

### Code availability

Code for R/Bioconductor^47^,^51^,^52^ packages is available at http://bioconductor.org. Custom R code and methods to calculate component-based PTGS scores is archived via the CERN OpenAIRE online service Zenodo (DOI: 10.5281/zenodo.570115).

### Data availability

Freely available data were used in the project throughout. Data sources included the Connectivity Map (CMap)^25^, NCI-60 DTP human tumour cell line screen database^30^, the Molecular Signatures Database (MSigDB)^50^, the Open TG-GATEs toxicogenomics database^16^, the Liver Toxicity Knowledge Base^33^ and Cmax and DILI potential-related information extracted from various studies^32^,^38^,^39^,^40^,^41^,^74^,^75^,^76^,^77^, as detailed in Supplementary Data 16. Validation data generated in the study is available at FigShare (DOI: 10.6084/m9.figshare.4954583). All other data are available on reasonable request.

## Additional information

**How to cite this article:** Kohonen, P. *et al*. A transcriptomics data-driven gene space accurately predicts liver cytopathology and drug-induced liver injury. *Nat. Commun.* **8,** 15932 doi: 10.1038/ncomms15932 (2017).

**Publisher’s note:** Springer Nature remains neutral with regard to jurisdictional claims in published maps and institutional affiliations.

## Supplementary Material

Supplementary Information

Supplementary Data 1

Supplementary Data 2

Supplementary Data 3

Supplementary Data 4

Supplementary Data 5

Supplementary Data 6

Supplementary Data 7

Supplementary Data 8

Supplementary Data 9

Supplementary Data 10

Supplementary Data 11

Supplementary Data 12

Supplementary Data 13

Supplementary Data 14

Supplementary Data 15

Supplementary Data 16

Supplementary Data 17

Supplementary Data 18

## Figures and Tables

**Table 1:** Key features of data processing that generated the Predictive Toxicogenomics Space (PTGS).

Data items*	**Number**†	**Percentage**‡
*Data Points*		
Entire Data set (CMap)	84 M	100
Data set after pre-processing of the most abundant platform (A)	34 M	41
Data set mapped to MSigDB-C2 gene sets (B1)	8 M	9.5
Data set mapped to the component model (B2)	0.6 M	0.7
PTGS scores calculated from the data set (D3)	3,062	0.004
		
*Instances*		
Entire Data set (CMap)	6,100	100
Instances after selecting one array platform, pre-processing and averaging (A)	3,062	50
Instances in the crossover data set with toxicity data (C1)	492	8
Instances with toxicity above GI_50_ (C2)	121	2
		
*Compounds*		
Entire Dataset (CMap)	1,309	100
Compounds after pre-processing of the most abundant platform (A)	1,217	93
Compounds with toxicity data (C1)	222	38
Compounds with toxicity above GI_50_ (C2)	68	5
		
*Components*		
Full component model (B2)	100	100
PTGS components (D3)	14	14
DILI predictive components (E5)	4	4
		
*Genes*		
Genes mapped to Ensembl IDs in the CMap HG-U133A series (A1)	11,948	100
Genes after pre-processing of the most abundant platform (A2)	11,350	95
Genes responding to chemical perturbations (A4)	6,064	51
PTGS associated genes (D5)	1,331	11
DILI predictive genes (E5)	299	2.5

^*^Steps in data reduction and analysis, letters refer to detailed explanations in Supplementary Fig. 1.

^†^M=1 million data points.

^‡^Percentages calculated from the first item in the category.
