# Supplementary material for: A transcriptomics data-driven gene space accurately predicts liver cytopathology and drug-induced liver injury
Source: Nat Commun. 2017 Jul 3;8:15932. doi: 10.1038/ncomms15932 (PMC5500850; doi:10.1038/ncomms15932)
Supplement: Supplementary Information [file ncomms15932-s1.pdf]

Supplementary Figure 1

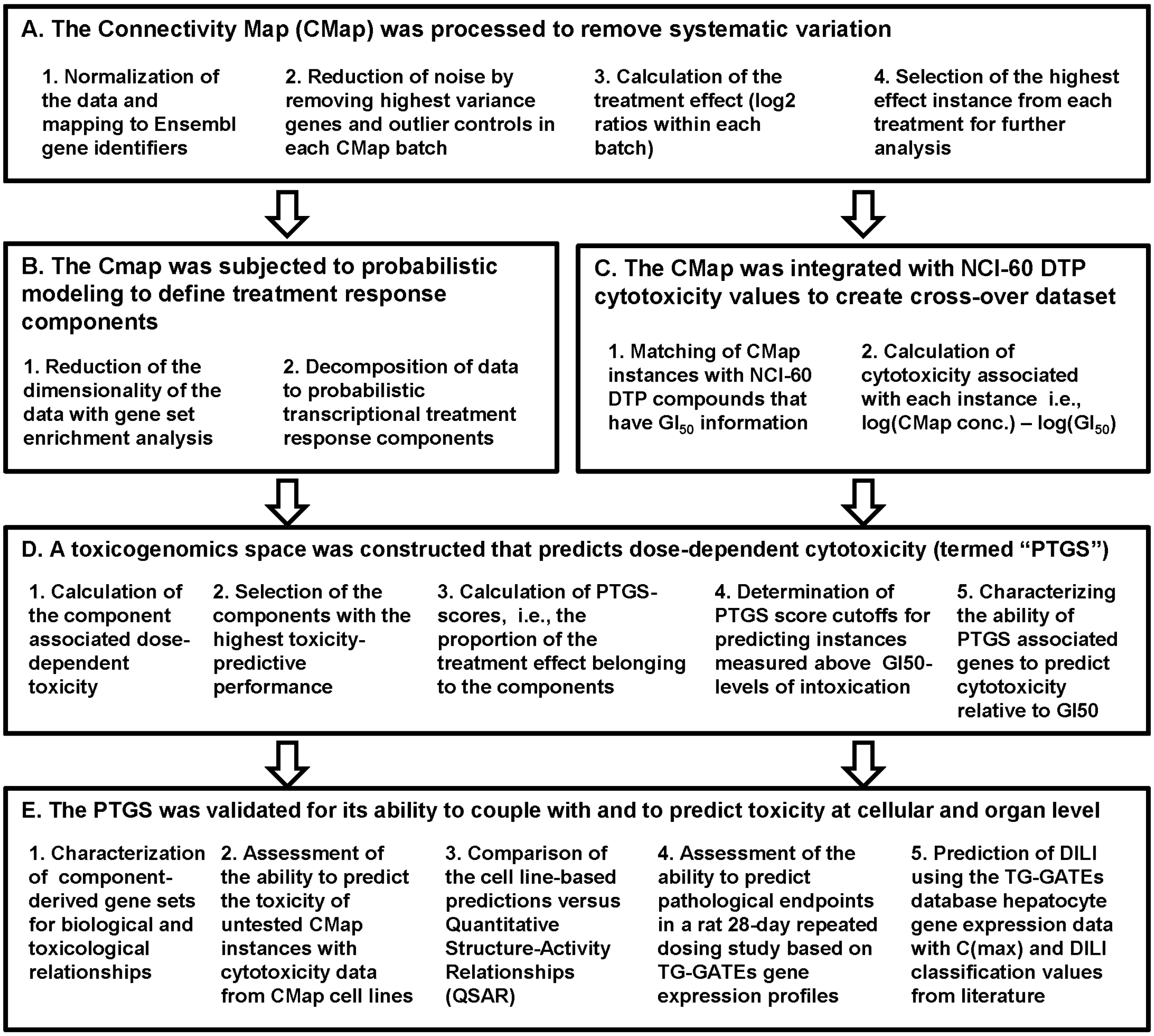

Supplementary Figure 1. Details of the component modelling and steps in generating and validating a PTGS. The steps (boxes) and the flow of information (arrows) through the modelling process, including four steps (A-D) to establish the predictive model for calculation of the PTGS scores and validation steps (E) are shown. (**a**) The Connectivity Map (CMap) data set is pre-processed to remove systematic variation. (**b**) Biological prior knowledge in the form of gene sets summaries is applied in conjunction with probabilistic modeling to decompose the transcriptional response space to minimally sized set components with interrelated activities. (**c**) Concentration-dependent cytotoxicity is obtained for the CMap instances by integrating with NCI-60 data; concentration that inhibited growth to 50% (GI_{50}) (**d**) Associations of the components to cytotoxicity are sought by evaluating their ability to predict concentration-dependent cytotoxicity for the instances with cytotoxicity data, using repeated cross-validation procedures. The Predictive Toxicogenomics Space (PTGS) is defined by the subset of components with an optimal cytotoxicity-predictive performance. Gene-based and model-derived methods are defined and validated separately. (**e**) Genes that are most active in each of the PTGS components are analyzed to help determine the biological mechanisms that characterize their ability to predict cytotoxicity. The PTGS score is further validated in vitro versus independent measurements from CMap and TG-GATEs databases and compared against quantitative structure-activity relationship analyses. TG-GATEs rat repeated dosing 29-day study data set is used to characterize the predictive ability of the PTGS in rat liver. Finally, the PTGS gene-based scoring concept is applied to the prediction of human drug-induced liver injury (DILI), i.e., a C_{max} concentration raising DILI concerns using a subset of the components (G, H, N and I).

Supplementary Figure 2

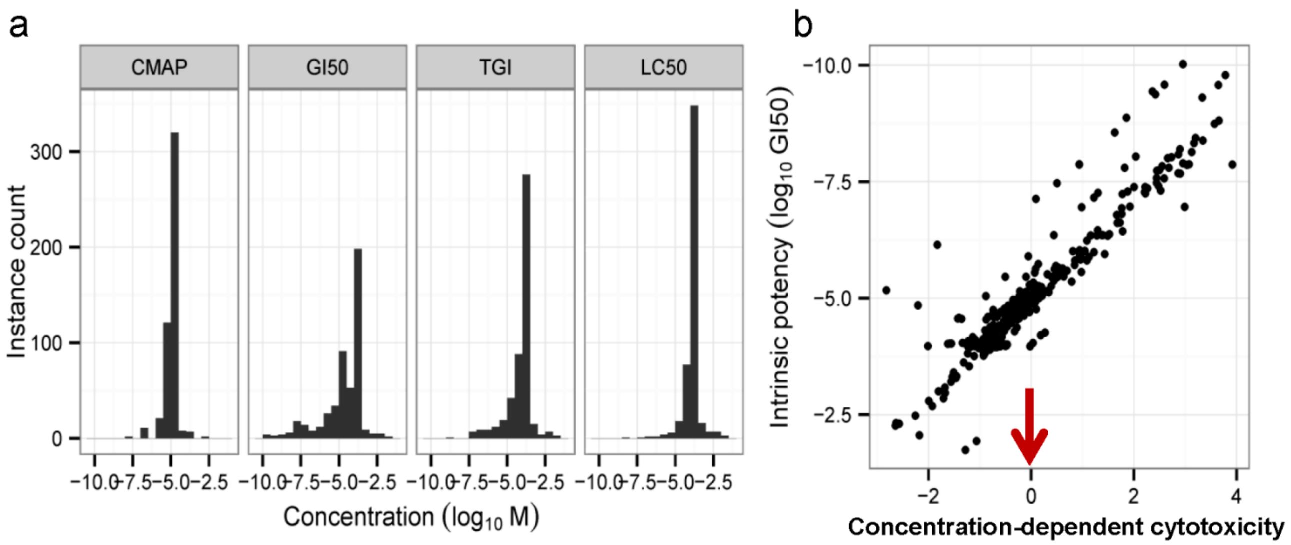

Supplementary Figure 2.The CMap and NCI60-DTP cross-over cytotoxicity data. (**a**) Histograms of the CMap and NCI-60 endpoint concentration values are shown, i.e., GI_{50} (50% growth inhibition), TGI (total growth inhibition), and LC_{50} (50% lethal concentration), for the 492 measurement instances. The same concentration (10 µM) was applied for most compounds. **(b)** The CMap instances show a very high correlation between the concentration-dependent cytotoxicity and intrinsic potency values (Pearson correlation 0.94); Concentration-dependent cytotoxicity (= *log_{10} CMap concentration* minus *log_{10} GI_{50} value*). These plots show that the CMap experimental design focused on the same dosage rather than similar levels of biological stimulation, i.e., the compounds with higher potency were dosed above their GI_{50} value, while compounds with lower potency were dosed below their GI_{50} value. This enabled modeling to cover a very wide range (~10^6-fold) of potencies around GI_{50}. For data see **Supplementary Data 1**.

Supplementary Figure 3

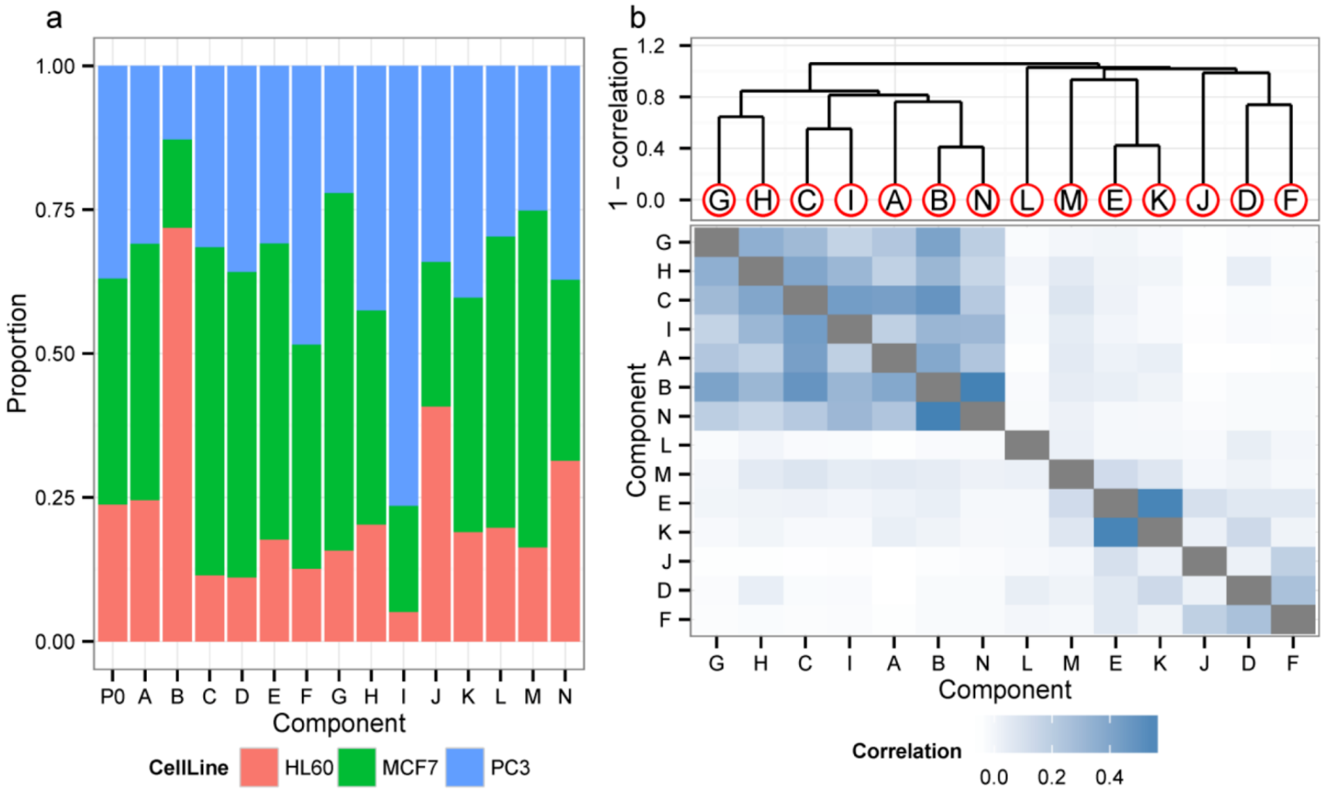

Supplementary Figure 3. Visualization of cell line coverage and biological correlations of the PTGS components. (a) Proportions denoting the relative sums of the PTGS component activities in different cell lines are shown. The *a priori* or expected proportions, as computed based on the numbers of instances in each cell line, are shown as *"P0"* on the left. The components are predominantly active to similar extents, besides components B and I which show over-representation relative to P0 in HL60 and PC3, respectively. **(b)** Hierarchical component-based clustering showing distance (or similarity) of the PTGS components, calculated using correlations between component activities in the CMap data set (3062 instances). The heat map reveals one clear cluster, another less clear cluster, and an outlier component (L).

Supplementary Figure 4

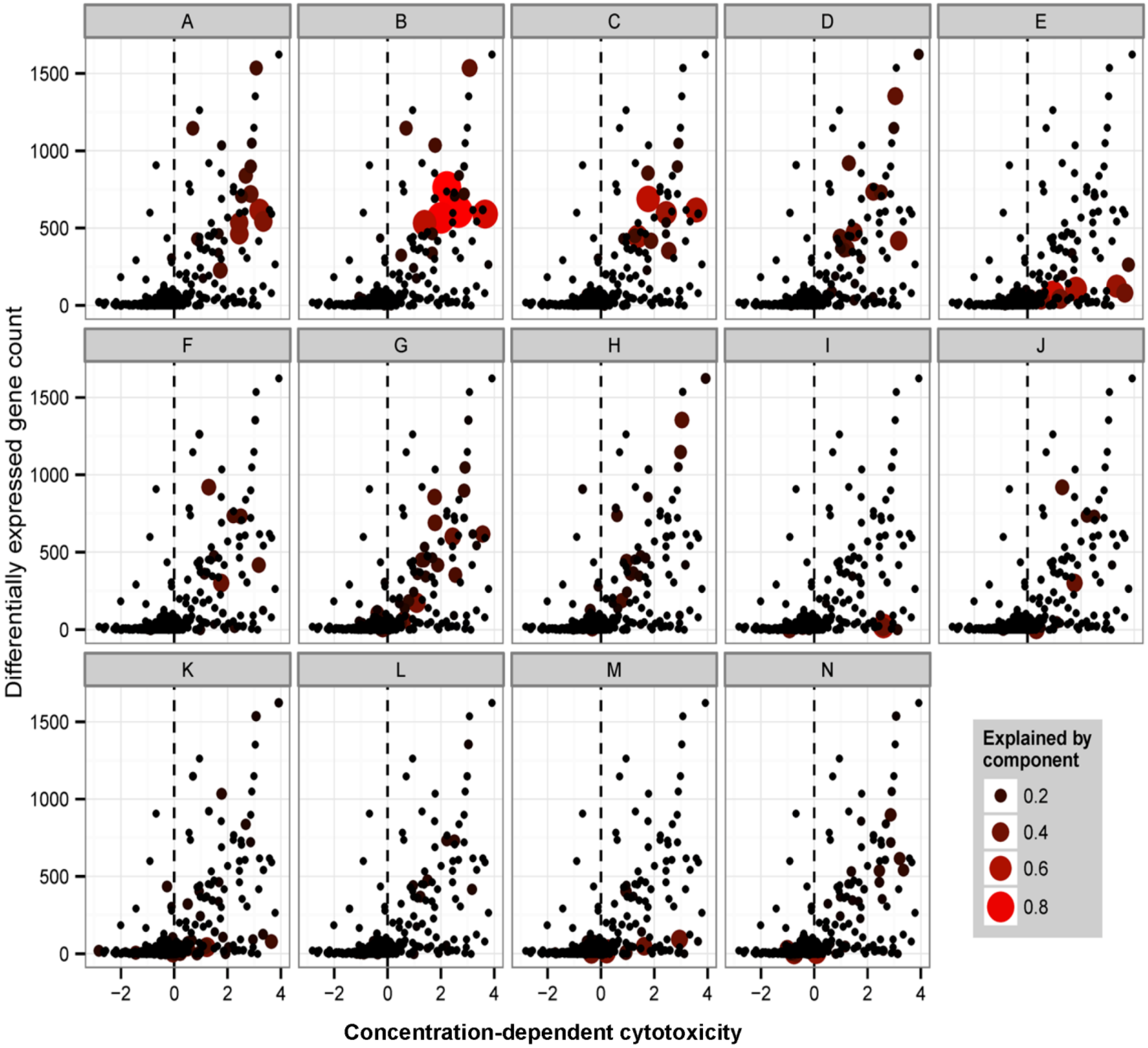

Supplementary Figure 4. Instance coverage of the PTGS components. Scatter plots show for the PTGS components (A-N) their individual coverage of the transcriptional variation of the instances (point size and colour indicates magnitude of the PTGS score, see Materials and Methods). The figure illustrates that the components cover instances within specific regions in the differential gene expression space versus the concentration-dependent cytotoxicity space. n=492. For data see **Supplementary Data 1**.

Supplementary Figure 5

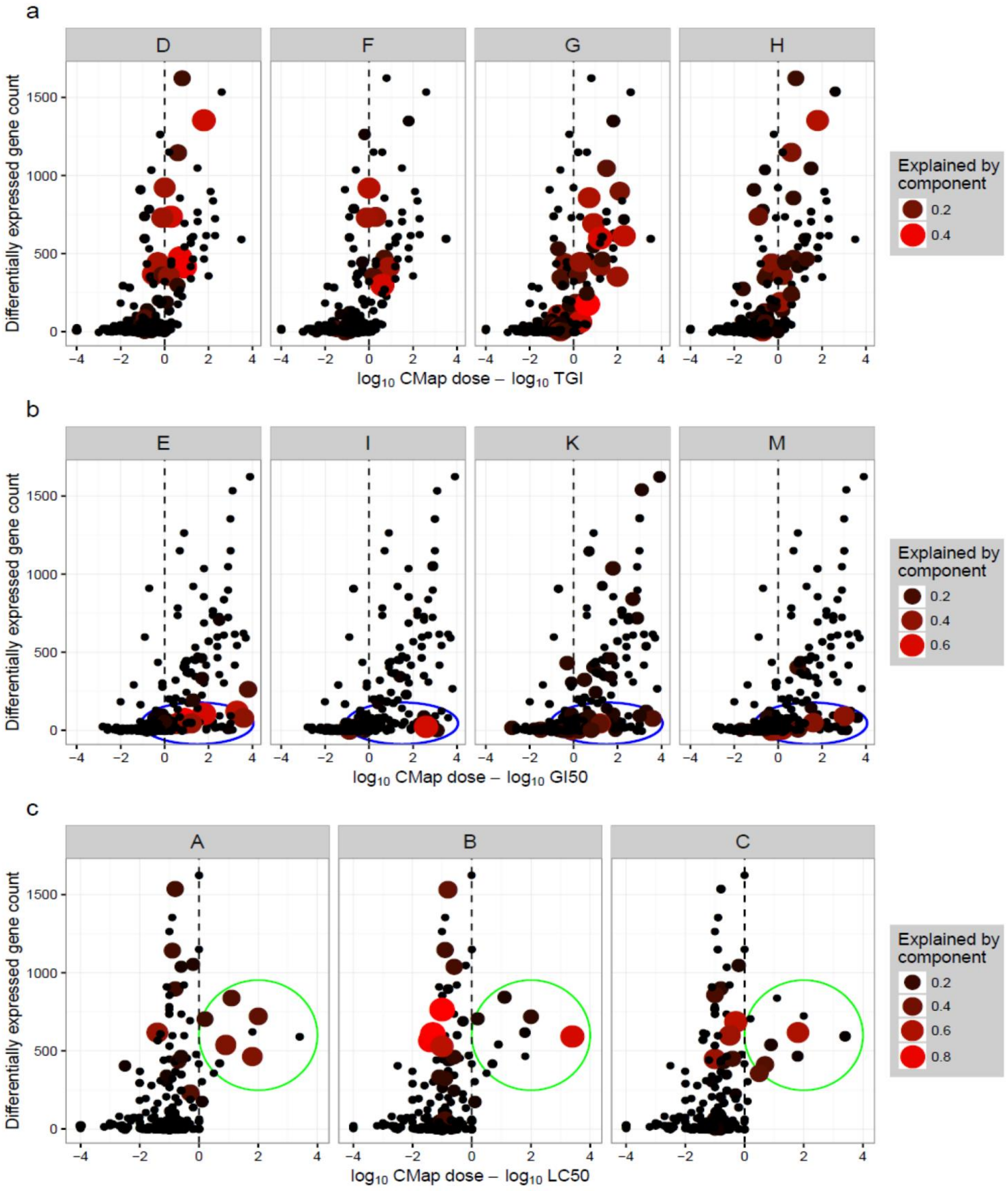

Supplementary Figure 5. Dose-response relationships of selected PTGS components illustrate the PTGS score response. (a) Components A-C become primarily active at cell killing doses i.e., above the LC_{50} level (green circle), cf. **Fig 2g** for the corresponding PTGS score data **(b)** The components D, F and J are active at or around the TGI-level (purple circle). **(c)** Components E, K and M become activated at above the GI_{50} level but below TGI (blue ellipse, cf. **Fig. 2c-d** for corresponding activation of the PTGS score at GI_{50} and TGI levels). The PTGS components therefore span a wide range of dose-responses around and above the GI_{50} level. N=492. For data see **Supplementary Data 1**.

Supplementary Figure 6

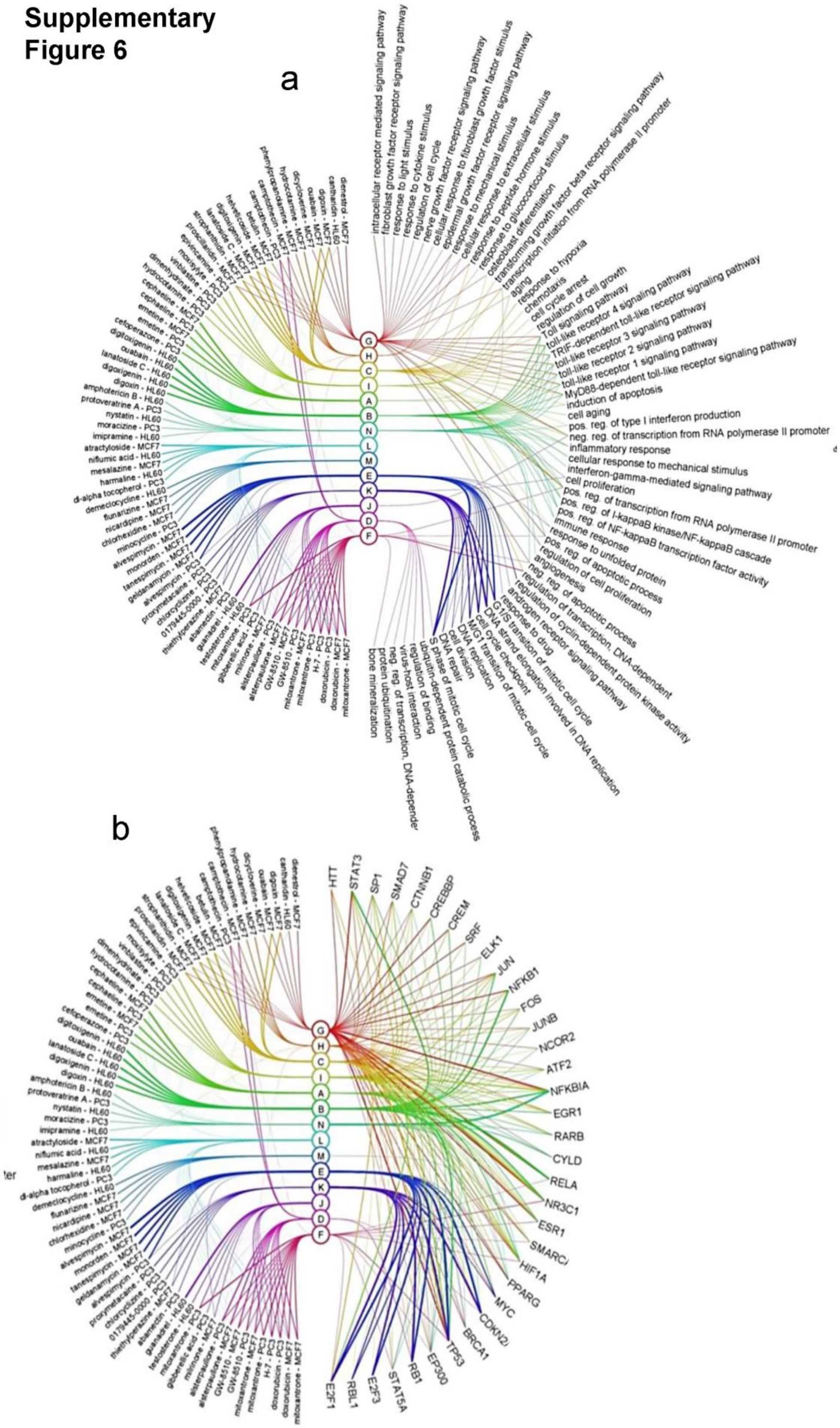

Supplementary Figure 6. Characterization of the PTGS components. “Eye diagrams” shows the associations between each of the 14 PTGS components (middle, colour) and the top five CMap instances (left) and top Gene Ontology Biological Process enrichments **(a)**, and upstream regulator enrichments **(b)**. Line widths indicate association strengths. The results reflect the clustering behaviour of the components (**Supplementary Fig. 3b, Supplementary Data 4 and 5).**

Supplementary Figure 7

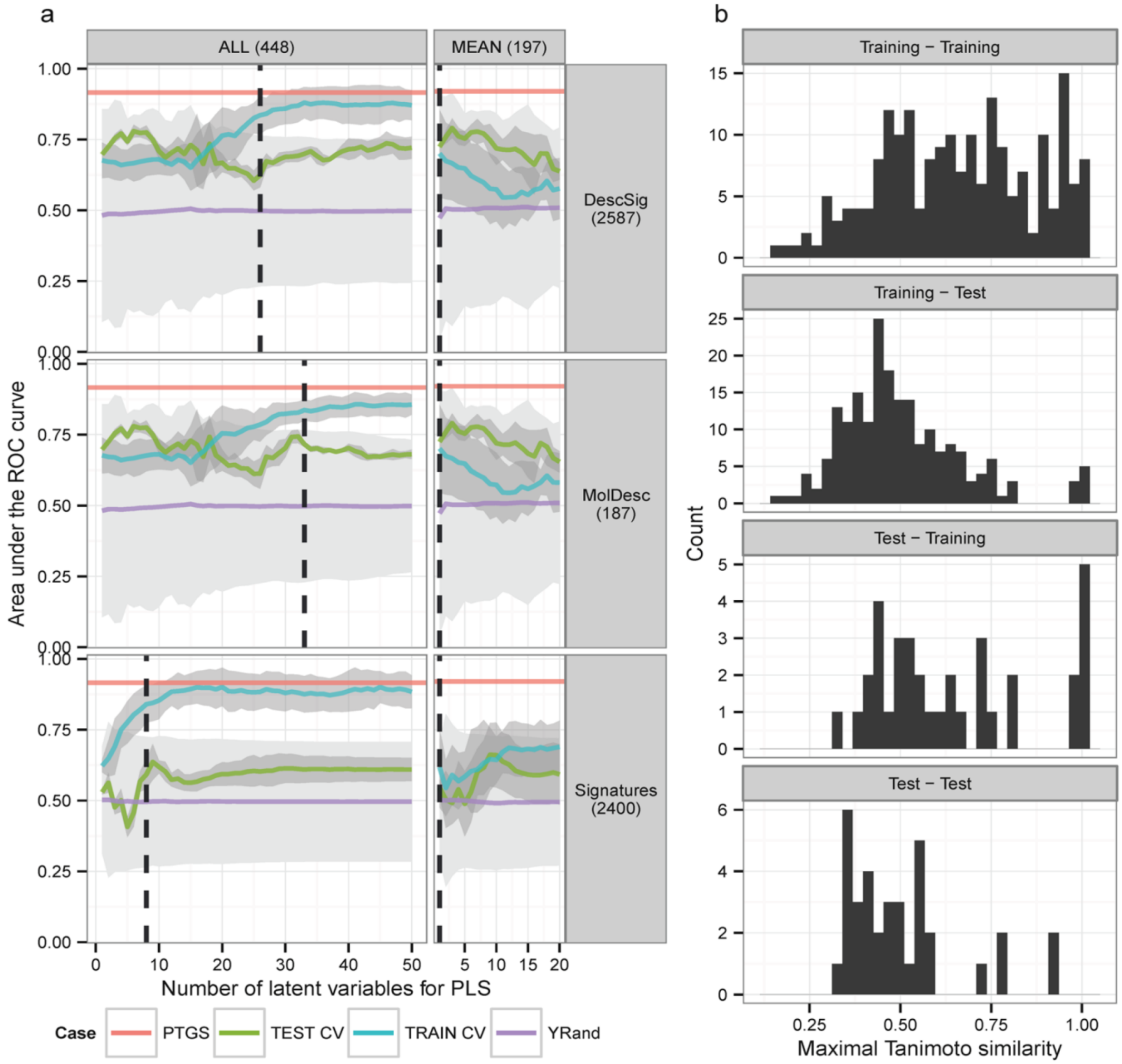

Supplementary Figure 7. Details of the compound structure-based analysis. (a) Partial Least Squares (PLS) Quantitative Structure-Activity Relationships (QSAR) results for two GI_{50} activity data subsets (ALL and MEAN; brackets = number of data points) for all instances concatenated (ALL) and instances averaged over the cell lines (MEAN). Three descriptor types were used: molecular descriptors (MolDesc), fragment-based descriptors (Signatures), and their combination (DescSig). AUC values are shown vs. the number of latent variables for PLS, internal cross-validation (TRAIN CV), prediction on test set (TEST CV) and y-randomized models on test set (YRand). Grey area indicates standard deviation over CV (TEST CV, TRAIN CV) or 95% confidence interval (YRand). The vertical dashed line indicates the number of latent variables that was selected based on CV. The results show that QSAR-based models to do not perform on the test set better than random models. **(b)** Distribution of maximum Tanimoto similarity (1.0 is identical) between a compound and the compound most similar to it. Compounds are compared within and between the QSAR Training and Test sets. The high structural diversity of compounds in the data sets likely explains the inability of the statistical models, which assume similar compounds to have similar toxicity, to find correlations between differences in compound structure and differences in mode of toxicity. Numbers in parentheses indicate the numbers of instances (x-axis) or features (y-axis) analysed.

Supplementary Figure 8

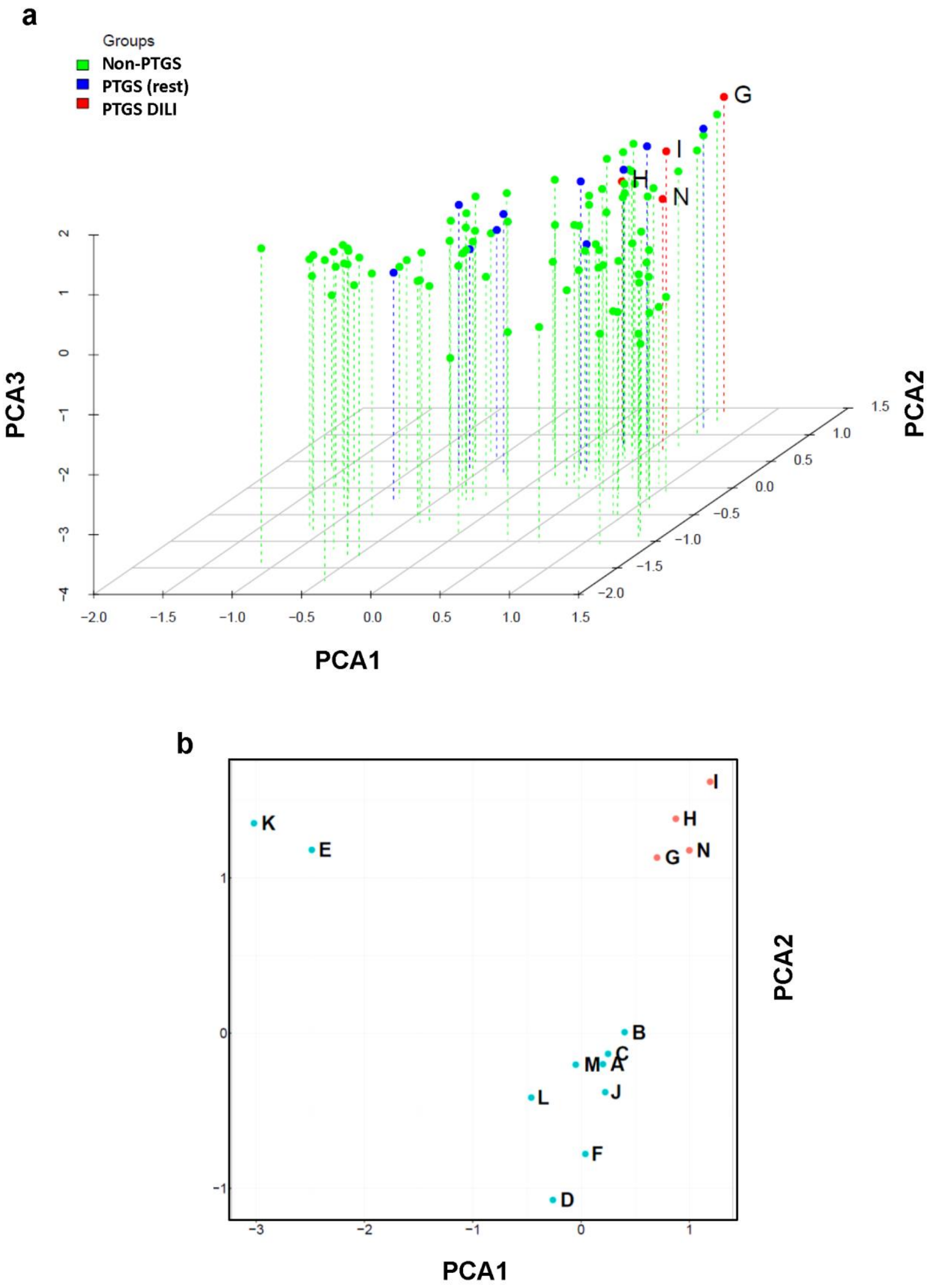

Supplementary Figure 8. Clustering of components based on principal components analysis with either (**a**) component activities in the CMap (100 components, 3062 instances) or (**b**) lists of PTGS-associated genes (1331 genes accross 14 components; **Supplementary Data 2**) Sets G, H, N and I co-cluster. Gene-based clustering is similar to the component-based, although it emphasizes the distinctness of the G, H, N and I cluster (red dots).

Supplementary Figure 9

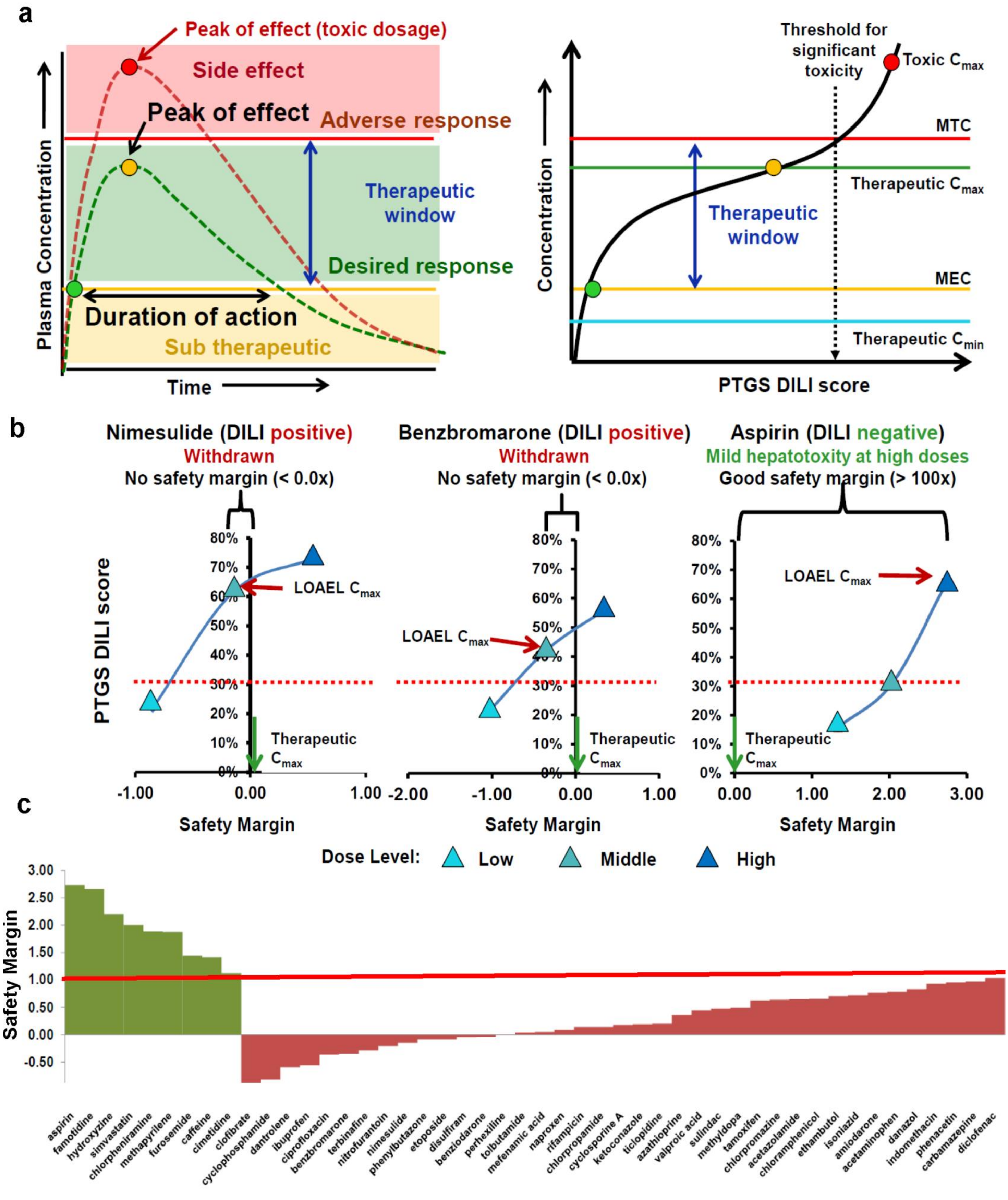

Supplementary Figure 9. PTGS predicts DILI by identifying concentration ranges without adverse effects. (a) The concepts of safety margin and therapeutic window illustrated and applied to the PTGS scoring concept. Abbreviations: MTC (maximum tolerable concentration); MEC (minimum effective concentration); Toxic C_{max} (peak concentration above the MTC). **(b)** Examples of predicting the safety margin using TG-GATEs rat hepatocyte profiles. PTGS DILI score (PropAct) = proportion of active DILI predictive genes. The gene sets from components G, H, N and I were applied to calculate the DILI score. LOAEL (lowest concentration where PTGS is above the threshold of significant activity i.e., PropAct > 0.3 and q<0.05) **(c)** Safety margins of positively predicted and DILI negative control compounds using TG-GATEs rat hepatocyte profiles measured at 8 hr. The approach predicts DILI with 71% sensitivity and 100% specificity (based on 64 compounds, 9 negative and 55 positive); Green bars (DILI negative), red (true positive i.e., correctly predicted); *Safety margin (log10) =* $log_{10}(LOAEL) - log_{10}C_{max}$. Red line: Safety Margin decision threshold is set to give 100% specificity of prediction based on the analysis of DILI negative controls, i.e., all of the negative controls are predicted to not elicit DILI concern at the therapeutic doses specified (*see Materials and Methods* and ***Supplementary Data 16-18*** for further details).
